# Precision environmental health monitoring by longitudinal exposome and multi-omics profiling

**DOI:** 10.1101/gr.276521.121

**Published:** 2022-06

**Authors:** Peng Gao, Xiaotao Shen, Xinyue Zhang, Chao Jiang, Sai Zhang, Xin Zhou, Sophia Miryam Schüssler-Fiorenza Rose, Michael Snyder

**Affiliations:** 1 Department of Genetics, Stanford University School of Medicine, Stanford, California 94304, USA;; 2 Life Sciences Institute, Zhejiang University, Hangzhou, Zhejiang 310058, China

## Abstract

Conventional environmental health studies have primarily focused on limited environmental stressors at the population level, which lacks the power to dissect the complexity and heterogeneity of individualized environmental exposures. Here, as a pilot case study, we integrated deep-profiled longitudinal personal exposome and internal multi-omics to systematically investigate how the exposome shapes a single individual's phenome. We annotated thousands of chemical and biological components in the personal exposome cloud and found they were significantly correlated with thousands of internal biomolecules, which was further cross-validated using corresponding clinical data. Our results showed that agrochemicals and fungi predominated in the highly diverse and dynamic personal exposome, and the biomolecules and pathways related to the individual's immune system, kidney, and liver were highly associated with the personal external exposome. Overall, this data-driven longitudinal monitoring study shows the potential dynamic interactions between the personal exposome and internal multi-omics, as well as the impact of the exposome on precision health by producing abundant testable hypotheses.

Human health is shaped by the personal genome, microbiome, and exposome (Topol 2014). Extensive studies have been conducted on the genome and microbiome; however, the human exposome is rarely investigated, especially at the individual level. Exposomics research aims to characterize all physical, chemical, and biological components collectively in the human external and internal environment and investigate the internal molecular, physiological, and health-related effects of these exposures (Gao and Snyder 2021). The external environment consists of all potential exposures from the near-field to the far-field sources of exogenous chemical, biological, and physical exposures (Li et al. 2012, 2014; Liu et al. 2019; Guo et al. 2020b). The internal environment includes but is not limited to xenobiotics and their biotransformation products, foreign DNA/RNA, foods along with the contaminants (Zhang et al. 2013; Jin et al. 2014; de Oliveira et al. 2018), and bioactive molecules accumulated from exogenous sources (Gao et al. 2018a; Vermeulen et al. 2020).

Compared with the human genome, the personal exposome is much harder to be decoded. Recent studies revealed that personal exposome profiles are highly dynamic and spatiotemporally different among individuals who live in the same geographical area. For instance, studies have shown that individuals are exposed to significantly different chemical and biological stressors during the same period even if they are in the same general geographical region, such as the San Francisco Bay Area or London (Jiang et al. 2018; Sinharay et al. 2018). Previous studies have usually targeted a single group of stressors, which failed to provide a holistic picture of the exposome cloud and their interactions (Gauthier et al. 2014). Moreover, stressor-induced physiological responses varied significantly among different individuals (Sinharay et al. 2018). Therefore, there is a critical need to monitor exposures at the individual level and systematically integrate them with respective internal multi-omics profiles to fully characterize each individual's personal responses to environmental exposures.

Multi-omics analyses enable a detailed investigation into the biological mechanisms underlying human phenotypes by integrating multiple omics, such as proteomics, metabolomics, and microbiomics (Gao 2021). Multi-omics profiling, together with clinical measures such as cytokines and blood tests, can comprehensively assess one's health status and detect significantly correlated exposures to understand the impact of the external exposome on human biology and health (Jiang et al. 2018; Schüssler-Fiorenza Rose et al. 2019; Zhou et al. 2019). In addition, longitudinal profiling can avoid biases introduced by one-time sampling and provide a molecular portrait of the effect of different exposures at an individual level.

In this first of its kind study, we used our previously published data sets to integrate thousands of longitudinally measured chemical and biological components along with physical factors in the personal exposome to investigate how the various stressors in the external exposome impacted internal -omes, such as the proteome, metabolome, and the gut microbiome, as well as cytokines and blood markers (Jiang et al. 2018; Schüssler-Fiorenza Rose et al. 2019; Zhou et al. 2019). Specifically, this study (1) improved the annotation of biological and chemical exposures in the external exposome and human blood; (2) integrated the external exposome with internal multi-omics to investigate the effect of the exposome on molecular phenotypes and pathways; and (3) correlated the environmental components with clinical measurements to associate the health effects of the external exposome.

## Results

### Longitudinal profiling of the exposome and internal multi-omics to monitor personal environmental health

**Figure 1.**
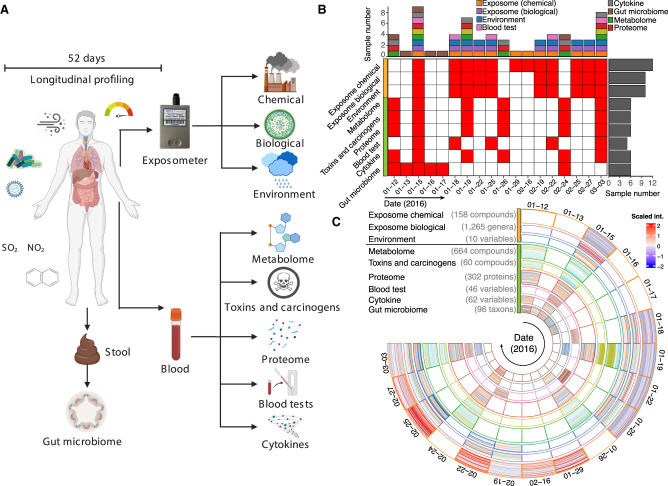
Overview of longitudinal sample collections for personal exposome and multi-omics profiling. (*A*) Personal exposome characterized by the exposometer includes environmental factors, biological components, and chemical stressors. Internal multi-omes include the gut microbiome, metabolome, proteome, toxins and carcinogens, cytokines, and blood tests. (*B*) The amount and collection time of each type of multi-omics and exposome samples. (*C*) Sample distribution and constitution of the exposome and internal multi-omics for monitoring precision environmental health.

We investigated whether the external exposome is associated with the internal molecular and physiology profile at a comprehensive and personal level using the schematic shown in Figure 1A. We first reanalyzed deep biological and chemical exposome data collected from our previously published study in which an individual had been continuously wearing a personal exposome collection device (exposometer), and we correlated the biological and chemical agents it captured with the internal molecular profiles. The individual in this study was a 61-yr-old male of European ancestry with doctoral education. He is a modest consumer of alcohol, does not smoke, is allergic to rubber and has very mild pollen allergies, and was prediabetic at the time of this study.

**Figure 2.**
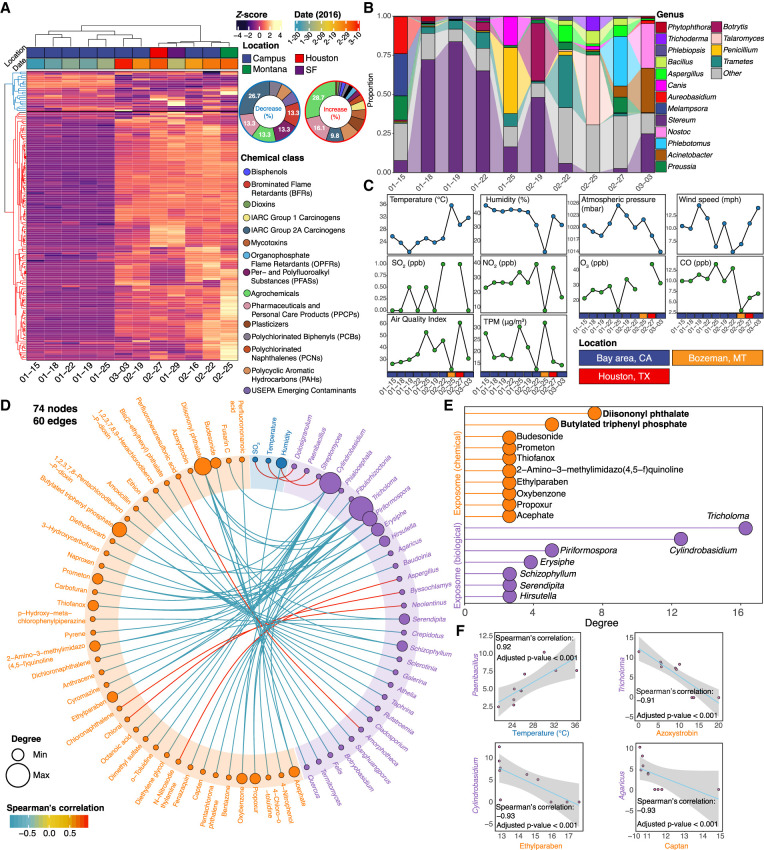
The dynamic and diverse personal exposome cloud. (*A*) Heat map clustering of the annotated chemical stressors in the exposome ordered by concentrations, with the sector diagrams indicating the increased and decreased chemical groups. The abrupt concentration increase after the January 25 sample indicates the approach can monitor changes of the chemical exposome. (*B*) Heat map of the top abundant genera annotated in the exposome during each collection period. (*C*) Environmental factors were collected by either the personal exposometer (temperature, humidity, total particulate matter) or local monitoring stations. (TPM) Total particulate matter. (*D*) Spearman's correlation analyses within the personal exposome (|r| > 0.9; false-discovery rate [FDR]–adjusted *P*-value [*Q*-value] < 0.05). (*E*) Chemical and biological components that have the most significant correlations with the other substances in the exposome. (*F*) Representative Spearman's correlation analyses between fungi and temperature/antifungal chemicals.

Over the 52-d period relevant for this study, the device captured organic chemicals using zeolite, followed by methanol elution and liquid chromatography coupled to high-resolution mass spectrometry (LC-HRMS) analysis. Biological specimens were also captured using polyethersulfone filters, and the DNA and RNA nucleic acids were analyzed by high-throughput sequencing (Supplemental Data S1). General environmental factors (e.g., temperature, humidity, total particulate matter) were recorded by the device, and the other environmental factors were also obtained from the local air quality monitoring stations (Fig. 2C). Contrary to conventional exposome monitoring studies, which usually focused on the exposures at a single time point (Gao et al. 2018b; Xiang et al. 2018a,b), we captured personal exposome profiles across 18 time points and annotated 1265 genera, 158 known chemical stressors among 3299 chemical features, and 10 environmental factors, which included physical stressors that may impact environmental health in this study (Fig. 1B). These genera and known chemical stressors were annotated from improved microbiome and chemical annotation pipelines that we developed as part of this study (Methods).

Over the same 52-d period, we also collected stool and blood samples from the same participant to profile the gut microbiome, proteome, metabolome, toxins and carcinogens, cytokines, and blood tests (Fig. 1C; Supplemental Data S1). Through reanalysis pipelines, we were able to annotate 60 toxins and carcinogens, as well as 664 metabolites, 302 proteins, and 62 gut microbiome taxa. We also measured 62 cytokines and 46 clinical blood parameters (Supplemental Data S1) to longitudinally monitor personal health status (Schüssler-Fiorenza Rose et al. 2019). All sample collections were performed during the first quarter of 2016 from three distinct locations in the United States (Supplemental Fig. S1). However, because not all sample types were collected at each time point, the inter-omic analyses were performed only when sample collection periods overlapped (Fig. 1B). Despite our limited ability to control all confounding variables, we searched for significant intra- and interexposome correlations and high-degree components (i.e., highly connected components) that have the most significant correlations in each analysis as those may play important roles in the interactions between the exposome and internal -omes (|r| > 0.9; FDR-adjusted *P*-value [*Q*-value] < 0.05). We tested various analysis methods (e.g., Pearson, Spearman's, Kendall, and partial correlations, Bayesian approaches, and mediation analysis) with different cutoffs and found the presented approaches and parameters were most suitable for these data sets with enough statistical power in terms of providing testable hypotheses (Methods).

### Intra-exposome relationships in the highly dynamic and diverse personal exposome cloud

To annotate as many chemicals as possible, we searched through the 3299 LC-HRMS raw features of the chemical exposome using various public exposome-related databases, as well as an in-house database that we assembled (Methods). Using this new annotation pipeline, we were able to annotate 158 known chemical stressors (Methods; Fig. 2A). These stressors were categorized into 13 classes, with the dominant class being agrochemicals, followed by pharmaceuticals and personal care products (PPCPs), plasticizers, and International Agency for Research on Cancer (IARC) group 2A carcinogens, and the chemicals in each class showed dynamic changes during the monitoring period (Fig. 2A; Supplemental Fig. S2; Supplemental Data S2). To characterize the biological exposome domain, we circumvented the limited ability of 16S rRNA/18rRNA/ITS sequencing by applying metagenomic sequencing. We found 17 genera dominated the study period, most of which were fungi and bacteria, and these also underwent dynamic changes (Fig. 2B). Ten general environmental factors, measured either by personal exposometer (temperature, humidity, and total particulate matter) or by local air monitoring stations (atmospheric pressure, wind speed, SO_2_, NO_2_, O_3_, CO, and air quality index), were also included in the study (Fig. 2C; Zhang et al. 2021).

We performed intra-omics correlation analyses to investigate the potential relationships among all the exposome components (Methods; Supplemental Data S3). We found a total of 60 statistically significant correlations (|r| > 0.9 and *Q*-value < 0.05) among 74 exposome components, including 41 chemicals, 30 genera, and three environmental factors (Fig. 2D; Supplemental Fig. S3). Specifically, diisononyl phthalate (a plasticizer) and butylated triphenyl (an organophosphate flame retardant) had the most significant correlations, followed by various agrochemicals, PPCPs, and IARC group 2A carcinogens. Among the biological components, the *Tricholoma* mushroom had the highest number of significant correlations, followed by *Cylindrobasidium*, *Piriformospora*, *Erysiphe*, *Schizophyllum*, *Serendipita*, and *Hirsutella*, all of which are fungi (Fig. 2E). In terms of environmental factors, only temperature and humidity collected by the exposometer as well as SO_2_ concentration collected by the local monitoring stations were significantly correlated with biological exposome components (Fig. 2D). For example, *Paenibacillus* was positively correlated with the temperature, consistent with the literature that members of *Paenibacillus* are heat resistant and grow well in warmer temperatures (Kaur et al. 2018). Azoxystrobin, ethylparaben, and captan are fungicides or antifungal agents (Rodrigues et al. 2013; European Food Safety Authority [EFSA] et al. 2020; Hu et al. 2022) that negatively correlated with different fungi (Fig. 2F). More details regarding the correlations within the exposome cloud are provided in Supplemental Data S2 and S3.

### Inter-omics analyses between the exposome and multi-omics revealed physiological links to the exposome

**Figure 3.**
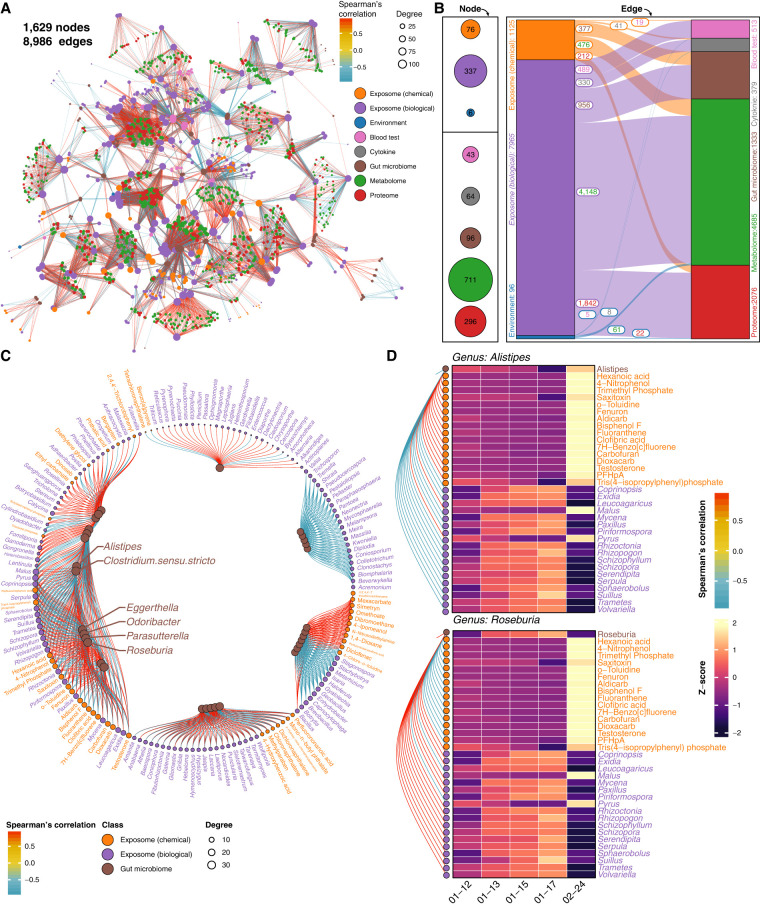
Precision environmental health network revealed by inter-omics analyses between the exposome and internal multi-omics. (*A*) Spearman's correlation network of all longitudinally profiled exposome and internal -omes. (*B*) Significant Spearman's correlations between the exposome and the internal multi-omics (|r| > 0.9; *Q*-value < 0.05). (*C*) Spearman's correlation analysis between the individual's exposome and the gut microbiome. Only gut bacteria with degrees > 20 are shown, and the highest-degree bacteria are named. The complete network is provided in Supplemental Figure S4C. (*D*) Heat map of the highest-degree personal gut bacteria and significant correlations with the exposome components (|r| > 0.9; *Q*-value < 0.05). Additional high-degree gut bacteria are provided in Supplemental Figure S4D.

To investigate how the exposome shapes an individual's phenome longitudinally, we investigated the links between the external exposome and internal multi-omics. Specifically, we found 8986 significant correlations (|r| > 0.9 and *Q*-value < 0.05) among 1700 factors from all -omes, and positive correlations were more predominant than negative correlations (Fig. 3A; Supplemental Data S4). The biological exposome and metabolome were the most extensive -omes in the network, and they also had the greatest number of significant correlations (N = 4148) (Fig. 3A,B). Additionally, we found that the exposome and internal multi-omics networks can be divided into several subnetworks with high modularity (0.819) (Supplemental Fig. S4A,B). More details regarding the correlations between the exposome and internal -omes are provided in Supplemental Data S4–S9.

### Personal exposome–gut microbiome interactions

We found 1333 significant correlations (|r| > 0.9 and *Q*-value < 0.05) between the exposome and the gut microbiome (16S rDNA data), and the number of positive and negative correlations were approximately equal (Fig. 3C). Specifically, the six highest-degree gut bacteria (each correlates with 34 exposome components) may be involved in multiple physiological processes that respond to the personal exposome. For example, members from *Alistipes* were shown to play essential roles in inflammation and various diseases (Parker et al. 2020); members from *Eggerthella* were implicated as the causes of liver and anal abscesses, ulcerative colitis, and systemic bacteremia (Lau et al. 2004); members from *Odoribacter* were found to maintain short-chain fatty acid availability and systolic blood pressure (Gomez-Arango et al. 2016); members from *Parasutterella* were involved in bile acid maintenance and cholesterol metabolism (Ju et al. 2019); whereas members from *Roseburia* played vital roles in producing short-chain fatty acids and anti-inflammatory pathways (Tamanai-Shacoori et al. 2017). Out of the top six genera, all but *Roseburia* positively correlated with chemical stressors and usually negatively correlated with biological components (Fig. 3D; Supplemental Fig. S4D). As a result, members from *Alistipes*, *Eggerthella*, *Odoribacter*, and *Parasutterella* were more likely to be involved in proinflammatory processes, whereas members from *Roseburia* were mainly involved in anti-inflammatory processes. On the exposome side, *Botryosphaeria*, *Corynespora*, and *Enterobacter* were the highest-degree genera (each correlated with 18 gut bacteria) among all exposome components, indicating their essential roles in interacting with the participant's gut microbiome (Fig. 3C). Overall, these results show an association of the external exposome with the gut microbiome and its associated biological processes, particularly inflammation.

### Exposome–proteome interaction network

**Figure 4.**
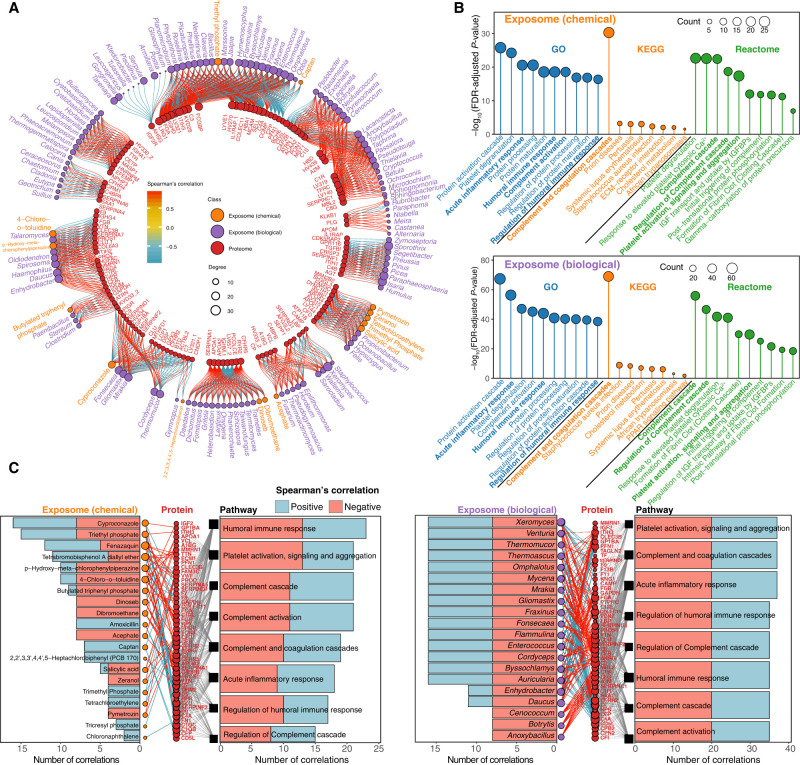
Exposome–proteome interactions: proteins and signaling pathways that were significantly correlated with the exposome. (*A*) Spearman's correlation analysis between the exposome and proteome (|r| > 0.9; *Q*-value < 0.05). Only the proteins with degrees > 5 are shown. The complete network is provided in Supplemental Figure S5. (*B*) Signaling pathways that significantly correlated with the exposome revealed by pathway analysis using KEGG, GO, and Reactome databases. Immune-related pathways are shown in bold. (*C*) Spearman's correlation networks between chemicals, top 20 biological exposome components, immune-related proteins, and signaling pathways (|r| > 0.9; *Q*-value < 0.05), with positive correlations shown in blue and negative correlations shown in red. A detailed network for each pathway is provided in Supplemental Figure S6.

We found 2054 statistically significant correlations (|r| > 0.9 and *Q*-value < 0.05) between the individual's exposome and internal blood proteome. Most of the high-degree exposome components were biological components, and positive correlations were slightly more frequent than negative correlations (Fig. 4A). Specifically, we found 11 highest-degree substances (nine genera and two chemicals), each of which was significantly correlated with more than 22 proteins in the proteome. The high-degree biological genera were fungi and primarily positively correlated with proteins, besides *Xeromyces*, which negatively correlated with proteins. Fenazaquin (a pesticide) and tetrabromobisphenol A diallyl ether (a brominated flame retardant) were two high-degree chemical stressors, both of which primarily negatively correlated with proteins, indicating some potential interactions among them. On the proteome side, 17 highest-degree proteins (each correlated with 21 exposome components) were discovered, and 14 of these are directly immune-related (Fig. 4B). For instance, alpha-2-HS-glycoprotein promotes endocytosis; complement C3 activates the complement system; and fibrinogen alpha chain is involved in both innate and T cell–mediated pathways. As such, these proteins primarily positively correlated with the external exposures in this study (Safran et al. 2010). Additionally, we discovered significantly correlated signaling pathways when queried against GO, KEGG, and Reactome databases (Supplemental Data S5, S6). Chemical and biological exposome shared several significantly correlated pathways, such as protein activation cascade, platelet degranulation, and acute inflammatory response, whereas some pathways were uniquely correlated with the chemical exposome, such as platelet activation, signaling, and aggregation pathway (Fig. 4B). Moreover, immune-related pathways were among the most common high-degree signaling pathways correlating with the exposome, with approximately half of those pathways positively correlated and the other half negatively correlated (Fig. 4C).

### Exposome–metabolome interaction network

The blood metabolome is considered the most interactive -ome with the exposome because xenobiotics interact with endogenous metabolites initially after entering the human body. In fact, the blood exposome overlaps with the blood metabolome from an analytical perspective as current approaches cannot distinguish the sources of the molecules present in the blood. Moreover, xenobiotic biotransformation is similar to that of metabolic pathways and can even involve the same enzymes, such as cytochromes P450 (Gao et al. 2018b; Walker et al. 2019). Therefore, it is essential to investigate the interactions between the exposome and metabolome to better understand the initial health impact of the exposome.

**Figure 5.**
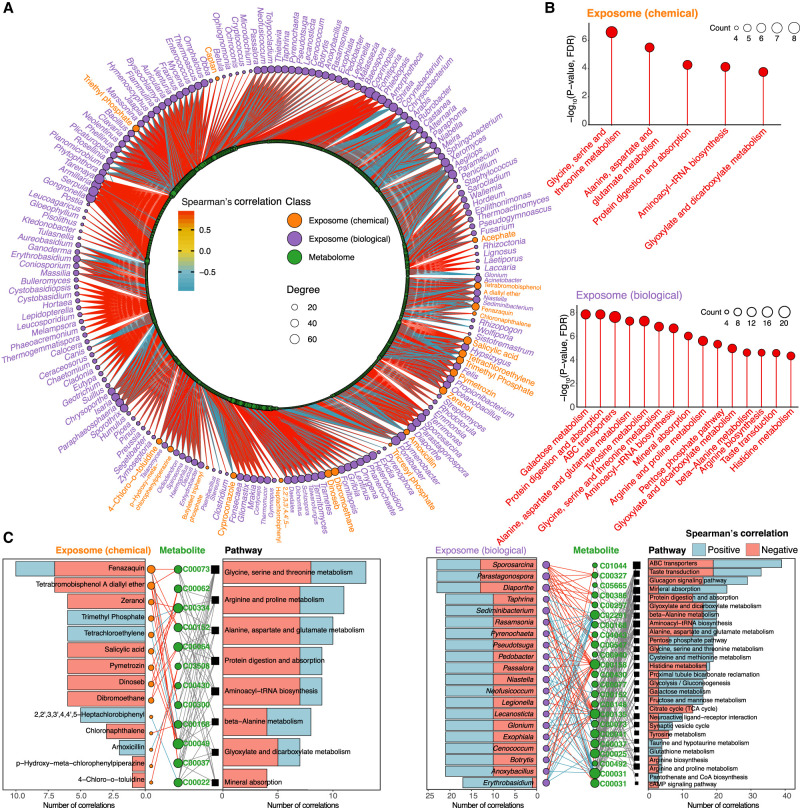
Exposome–metabolome interactions: metabolites and metabolic pathways that were significantly correlated with the exposome. (*A*) Spearman's correlation analysis between the exposome and metabolome. (*B*) Significantly correlated metabolic pathways revealed by pathway analysis using the KEGG database (|r| > 0.9; *Q*-value < 0.05). (*C*) Significant correlations between chemicals and top 20 biological exposome components and metabolites (represented by KEGG compound entry) and metabolic pathways revealed by Spearman's correlation networks (|r| > 0.9; *Q*-value < 0.05), with positive correlations shown in blue and negative correlations shown in red. The complete network is provided in Supplemental Figure S8.

We found 4624 statistically significant correlations (|r| > 0.9 and *Q*-value < 0.05) between the exposome and internal blood metabolome. Positive correlations were more frequent in the exposome–metabolome analysis than the exposome–proteome analysis (Fig. 5A; Supplemental Fig. S5). The high-degree biological components were primarily fungi and usually positively correlated with the metabolites; exceptions are *Aegilops* (a grass), the bacteria *Pontibacter* and *Hymenobacter*, and *Paramecium* (a ciliated protist). Salicylic acid (a PPCP), dinoseb (an herbicide), and dibromoethane (an IARC group 2A carcinogen) were the three highest-degree chemicals, all of which primarily positively correlated with endogenous metabolites. Overall, we found 19 high-degree metabolites, each significantly correlated with 21 exposome substances. Several metabolic pathways were significantly correlated with both the chemical and biological exposome (Methods; Supplemental Fig. S7), such as protein digestion and absorption and aminoacyl-tRNA biosynthesis, whereas some pathways were only correlated with the biological exposome (Fig. 5B; Supplemental Data S7, S8). Similarly to the exposome–proteome analysis, we performed correlation network analysis among the exposome, metabolites, and metabolic pathways. Trimethyl phosphate (a plasticizer and organophosphate flame retardant) and tetrachloroethylene (an IARC group 2A carcinogen) were positively correlated with all the metabolic pathways, whereas tetrabromobisphenol A diallyl ether, salicylic acid, and zeranol (a mycotoxin) were negatively correlated with all metabolic pathways (Fig. 5C). Thus, a diverse array of external exposures was associated with various internal metabolic and proteomic changes.

### Monitoring precision environmental health by investigating the exposome–clinical data correlations

**Figure 6.**
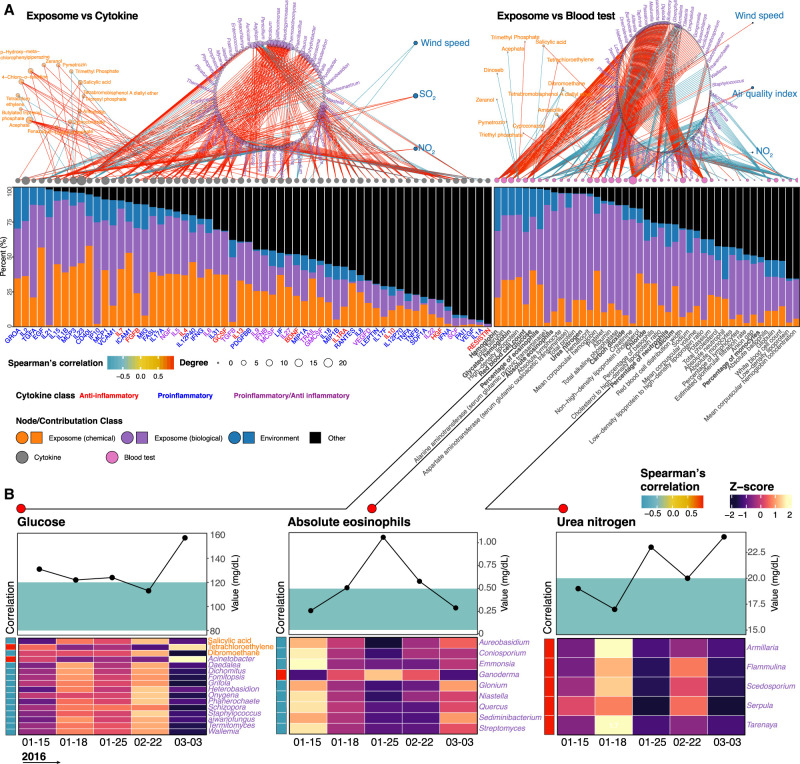
Effects of the exposome on precision environmental health. (*A*) Relative contributions of various exposome components on the alterations of personal cytokines and blood tests (|r| > 0.9; *Q*-value < 0.05). Bold blood tests had out-of-reference values. (*B*) Representative blood test results with corresponding reference ranges (green areas) and their significantly correlated exposome components.

Standard clinical measurements such as blood and cytokine tests directly reflect the individual's health and are ideal indicators to investigate the health impact of the exposome. Based on our exposome–cytokine analysis, the biological exposome had the most significant correlations with cytokines, followed by chemical stressors and environmental factors: 362 significant correlations (|r| > 0.9 and *Q*-value < 0.05) were found between the exposome and cytokines, most of which were positive correlations. After converting correlation coefficients to variable importance in projection scores, we determined the contributions of all significantly correlated exposome components on cytokines (Methods; Supplemental Data S9). Specifically, 60% of the cytokine variation was explained by the determined factors in this study. Furthermore, the top 13 cytokines, which were almost entirely contributed by the annotated exposome components (>90%), were all proinflammatory cytokines (e.g., IL23 and VCAM1), indicating that these cytokines may play essential roles in response to the exposome. Additionally, 14 highest-degree (each correlates with more than seven exposome components) cytokines were found to be primarily positively correlated with the exposome (Fig. 6A). The most high-degree biological components were fungi, such as *Wallemia*, which are filamentous food-borne pathogens (Zajc and Gunde-Cimerman 2018). Moreover, other than *Xeromyces*, most of the exposome components were primarily positively correlated with cytokines, consistent with the exposome–proteome analysis, whereas only *Xeromyces* primarily negatively correlated with the proteins among high-degree biological components. Acephate, an insecticide, is the highest-degree chemical component, positively correlated with 10 cytokines (Fig. 6A).

Similar to the exposome–cytokine analysis, biological components had the most significant correlation with clinical blood tests, followed by chemicals and environmental factors. However, fewer chemicals were correlated with blood tests than those correlated with cytokines (Fig. 6A); 513 significant correlations were found between the exposome and blood tests, and the majority were positive correlations. Using similar contribution determination algorithms, 77% of the blood tests variation was explained by the determined factors in this study. The top 13 blood tests were almost entirely contributed by the determined exposome components (contributions of the exposome > 95%) and were primarily related to the immune system, liver, and kidney functions. Additionally, eight highest-degree (each correlated with more than 25 exposome components) clinical blood tests were primarily positively correlated with the exposome, but platelet was primarily negatively correlated. The highest-degree blood test, creatinine, which is a biomarker for kidney function, correlated with 62 exposome components (Fig. 6B). Unlike cytokine profiles, for which reference ranges have not been well established, blood tests have clinically established reference ranges. We therefore performed correlation analyses to understand the effects of the exposome on personal health using blood test results with out-of-reference values (Fig. 6A). We found out-of-reference values of blood glucose levels were significantly correlated with three chemical stressors and 13 microbes. For instance, salicylic acid concentration was negatively correlated with glucose level; salicylic acid has been previously shown to decrease glucose concentration and used as a treatment for type 2 diabetic patients (Rumore and Kim 2010), consistent with our findings. Similarly, out-of-reference values of absolute eosinophils and urea nitrogen correlated with specific biological exposome components, including some known pathogens, such as *Aureobasidium*, *Niastella*, and *Scedosporium* (Fig. 6B). Previous studies were consistent with our results as eosinophilic phagocytosis consumes eosinophils during allergy and inflammation (Shamri et al. 2011), and various pathogenic microbes can use urea as a nitrogen source (Rutherford 2014). Thus, the results show the feasibility of directly relating external exposures to clinical measurements at an individual level.

## Discussion

It has long been acknowledged that environmental factors affect personal health, but conventional environmental health studies face limitations. For example, (1) population studies may overlook the significant differences between individuals; (2) single time point sampling fails to reflect the chronic effects of stressors; and (3) focusing on a single or a class of stressors does not profile the holistic health impact of the exposome. To overcome these challenges, we generated a comprehensive precision environmental health profile by longitudinally monitoring both the personal exposome and internal multi-omic profiles (Fig. 1). In addition, we also measured standard clinical indices to investigate the potential health effects of the exposome. Using Spearman's correlation analysis, we discovered many significant correlated physiological parameters and exposome components, indicating their interactions in the participant's responses to the personal exposome. Although inferences from a single individual over a relatively short period are limited by biologic unknowns, our study provides vast testable hypotheses to further investigate the underlying mechanisms using analytical and experimental approaches. Overall, we found thousands of external chemical and biological exposures associated with the internal microbial, proteomic, and metabolic alterations within this individual, indicating a strong association between the external exposome and molecular health.

We were able to capture more than 3000 chemical features but only annotated 158 known chemical stressors by a broad annotation method that uses various databases, including those containing emerging contaminants (Li et al. 2017b; Xiang et al. 2017; Gao et al. 2019b,c). This indicates that existing exposome databases still lack the power to annotate the majority of the chemical exposome. The results showed that the concentrations of most chemical stressors increased after January 25, 2016, when the individual transitioned from a period of residing at home to a period of travel, indicating that the chemical exposome was greatly impacted by the individual's spatial changes (Supplemental Fig. S2F). Also, each location may have its unique chemical composition pattern. For instance, relatively high concentrations of diethyltoluamide (a personal care product) and tricyclazole (a pesticide) were found during travel to Montana, whereas relative high concentrations of hexanoic acid, trimethyl phosphate, and benzoic acid (a personal care product, a plasticizer, and a pesticide, respectively) were found during travel to Houston. However, brominated flame retardants were barely detected during these two travels compared with the exposures in San Francisco Bay Area (Fig. 2A; Supplemental Data S1). Agrochemicals had the highest concentrations among all annotated chemicals, indicating their ubiquitous presence in the environment. An alternative view is that agrochemicals are the most frequently studied chemical stressors, making them most easily identifiable. It is also worth noting that high concentrations do not necessarily imply high health risks because each chemical has its own safe dose, and the combined effects among them are still unclear (Gao 2021).

The biological exposome revealed a number of patterns as well. The fungal genus *Stereum* was dominant at most time points, reflecting its high abundance in the personal exposome during the monitoring period. However, when the individual traveled to Montana and Houston, *Talaromyces* and *Phlebotomus* became the most dominant genus, respectively. Both *Stereum* and *Talaromyces* are fungi, whereas *Phlebotomus* is a group of sand flies. This indicated that the biological exposome was highly impacted by geographical changes like the chemical exposome (Fig. 2B). Although the composition of the personal exposome significantly changed with travel locations, the internal biomolecules did not significantly change before and after the travel, indicating the relative stability of the internal biological system and the necessity of monitoring the external exposome (Supplemental Data S1). We also found several correlations of the biological exposome with chemical and environmental factors, such as associations of antifungals with a decrease in fungal exposures. Many of these are intended food or soil antifungal products (azoxystrobin and captan), whereas others are common preservatives (ethylparaben and anthracene) that have antifungal properties (Fig. 2F; Jiang et al. 2018; Sinharay et al. 2018). Overall, nearly 100 significant correlations were found by intra-exposome analysis, representing the complex interactions within the exposome domains. Specifically, the negative correlations of fungi with various pesticides and herbicides indicate these agrochemicals may inhibit the fungi growth as well (e.g., *Tricholoma* vs. propoxur and *Erysiphe* vs. bentazone). Moreover, we found several tertiary relationships, such as the mycotoxin fusarin C (produced by *Fusarium*) negatively correlated with *Cylindrobasidium,* suggesting a possible competition among the different fungi (Supplemental Data S3).

A recent study identified radioprotective gut microbes and internal metabolites in mice using a multi-omics analysis (Guo et al. 2020a), showing the potential of this approach to investigate essential components in the internal -omes that respond to the external environment. To this end, we performed inter-omics analyses between the exposome and gut microbiome, proteome, and metabolome, respectively. By discovering high-degree components in each analysis, we identified the critical components in the exposome-internal -omes interactions. For instance, we found the general environmental indicator such as air quality index was significantly correlated with the individual's creatinine, aminotransferase, cysteinylproline, monoglyceride, adiponectin, and immunoglobulin lambda (Supplemental Data S4). In addition, we found six highest-degree gut bacteria that may be important in the responses to the personal exposome. The high-degree biological components in both exposome–proteome and exposome–metabolome analyses were mainly fungi, yet few had known human health effects. However, we identified major high-degree annotated chemicals that are known human stressors; for instance, exposure to the herbicide dinoseb causes various developmental toxicities and loss of thyroid and body weight (Matsumoto et al. 2008), and brominated flame retardants like tetrabromobisphenol A diallyl ether are known neurotoxicants (Eriksson et al. 2001). In addition to the endogenous metabolites, we were able to annotate 60 toxins and carcinogens in the individual's blood based on the exposome-related databases (Fig. 1C). Unlike the annotated xenobiotics in the exposome samples, most of the chemical stressors annotated in the blood were food and animal toxins. Furthermore, only 11 chemical stressors were annotated in both the external and blood exposome. This is likely to be partly due to the limited power of the current databases, because most of the databases only contain the information of parent chemicals but not their biotransformation products (Gao 2021; Xu et al. 2022). Additionally, persistent hydrophobic substances tend to accumulate in adipose tissues but not in human blood, which we profiled, whereas nonpersistent hydrophilic chemicals are efficiently excreted out of the human body, limiting their detection (Gao 2021; Gao et al. 2018b). Finally, the bioavailability of chemicals in different external matrices also limits the exposure, dose-response, and concentration of bioavailable fraction (Li et al. 2017a; Zhang et al. 2017; Gao et al. 2019b). These results further illustrate the necessity of monitoring the external exposome rather than monitoring the internal exposome alone.

Overall, our results indicate that the immune system, kidney, and liver of this individual may play essential roles in response to the exposome, which are all known to regulate and respond to foreign substances (Shibutani et al. 2015; Bajaj et al. 2018; Xiang et al. 2018a). In the exposome–proteome analysis, we found 14 out of 17 high-degree proteins were involved in immune responses (e.g., complement C3, interleukin 1 receptor accessory protein, and immunoglobulin heavy chain proteins) (Shibutani et al. 2015), and immune-related pathways (e.g., acute inflammatory response, humoral immune response, and complement and coagulation cascades) were the major highest-degree signaling pathways. In the exposome–metabolome analysis, we identified 19 highest-degree metabolites related to protein metabolism, inflammation, and kidney and liver functions (e.g., L-arginine, nutriacholic acid, epsilon-[gamma-glutamyl]-lysine, and uracil), indicating that these metabolic pathways may be involved in responses to the exposome. Moreover, certain high-degree metabolic pathways were involved in both protein and immune-related pathways, such as alanine aspartate and glutamate metabolism, protein digestion and absorption, and beta-alanine metabolism. Specifically, specific protein metabolism pathways (e.g., amino acids synthesis and protein breakdown) were highly sensitive to oxidative stresses caused by the exposome components (Peters et al. 2021); inflammation is often the first immunological response to foreign substances; and the kidney and liver are the main detoxification organs (Peters et al. 2021), with liver and bile acids serving essential roles in responding to the foreign substances (Orešič et al. 2020).

To further investigate the potential health effects of the exposome, we performed Spearman's correlation analysis with cytokines and blood test results. Proinflammatory cytokines were the most significantly correlated with the external exposome components (e.g., IL23 and IL2), and they have been previously shown to be elevated after exposure to external stressors (Schaue et al. 2012). Our clinical blood test results provided further evidence as alterations in creatinine and urea nitrogen, biomarkers of kidney and liver functions, respectively, were correlated with specific exposome components. As a result, exposome–proteome analysis cross-validated with exposome–cytokine analysis, indicating that the inflammatory processes may play essential roles in responding to the exposome, and the exposome–metabolome analysis cross-validated with exposome–blood tests analysis, showing that the liver and kidney may play significant roles in responding to the exposome. Furthermore, the exposome–microbiome analysis showed that the highest-degree gut bacteria are related to the proinflammatory processes and liver metabolism. Therefore, these physiological processes and organs may be ideal candidates for testing the combined effects of multiple stressors in future studies.

On the exposome side, high-degree exposome components that overlapped in more than one inter-omic analysis are significant health concerns. Specifically, *Isaria*, *Sporothrix*, and *Tarenaya* were among the highest-degree microbes correlated with all internal -omes. Members of these genera were found to be involved in complex physiological mechanisms and may show adverse health effects. For example, species of *Isaria* were found to induce cell death (Chhetri et al. 2020); species of *Sporothrix* triggered skin and lung inflammatory reactions (de Lima Barros et al. 2011); and the pollen of *Tarenaya* members generate immunoglobulin E–mediated allergic reactions (Danella Figo et al. 2019). In addition, certain types of agrochemicals, PPCPs, and flame retardants that we detected are high-degree chemical stressors known to cause epigenetic alterations, endocrine disruption, impaired nervous system function, oxidative stress, and inflammation (Peters et al. 2021). Therefore, those substances would be ideal candidates for investigating the underlying mechanisms of their combined health effects in future studies. Such information may be especially important for understanding environmental triggers for inflammatory diseases such as inflammatory bowel diseases, autoimmune diseases, and skin inflammation. Finally, the presented integration approach provides the possibility to annotate novel health-related chemicals by correlating all the chemical features with the internal multi-omics and discovering which chemical features are high degree. By investigating the mass spectrum of these high-degree but unknown features, we might be able to discover novel health-related chemicals that are highly and extensively associated with the internal biomolecules. However, because of the obstacles of annotating unknown features by the current algorithms, we did not perform such analyses in this study, but they can be performed upon further annotation advances.

In conclusion, we found both time and location impacted the personal exposome, especially the biological components and environmental factors (Supplemental Fig. S2F), and the biological and chemical exposome were highly dynamic. In addition, the pathways related to the immune, liver, and kidney systems were highly associated with the external exposome. Because of the limitations of studying a single participant, rapid changes of the exposome and internal multi-omics, relatively low confident annotation power, and possible false-positive correlation in this study, future research should longitudinally and frequently monitor the precision environmental health of more individuals by using the approaches pioneered in this study and increase the annotation confidence of the chemical and biological components in the exposome. Moreover, because most of the current exposome databases do not contain experimental tandem mass spectrum (MS^2^) information and some stable environmental chemicals cannot generate MS^2^ with electrospray ionization probe, we did not filter out the annotated features with relative lower confidence levels. In fact, the features that have relatively lower confidence levels are also very valuable as testable hypotheses especially after we found they have the potential biological and environmental significance. Moreover, because the annotations of proteome and metabolome have higher confidence levels than the exposome, the high-degree proteins and metabolites are reliable even with certain false-positive correlations or annotations. Because the purpose of this pilot case study is to determine the feasibility of comprehensively integrating personal exposome and internal multi-omics to investigate the phenotypic variations, the validation of those testable hypotheses should be performed after this study. In addition, we focused on the airborne exposome and did not measure other exposures, such as dermal and ingestion exposures, inorganic chemical components (Gao et al. 2019a; Xu et al. 2019, 2020; da Silva et al. 2020), psychosocial stressors, and personal lifestyle, which may affect the clinical measurements as well (Fig. 6A). Nonetheless, this study shows the power of using a holistic approach of monitoring the exposome on personal environmental health using inter-omics analyses and serves as a useful approach to scale to the other individuals and locations. Our study also identified high-degree components as essential components among the exposome-internal -omes interactions and provided abundant testable hypotheses to further investigate their underlying mechanisms of impacting individual health.

## Methods

### Exposome sample collection

The participant in the study is enrolled under Stanford University's IRB protocols IRB-23602 and IRB-34907. The modified RTI MicroPEM V3.2 personal exposure monitor (RTI International), termed exposometer, was used to collect chemical and biological components exposed by individuals from January 2016 to March 2016. In addition, temperature, humidity, and total particulate matter were simultaneously collected by the exposometer in a real-time manner. The original sequential oiled frit impactor was removed to maximize the collection of biological components. A 0.8-mm pore-size polyethersulfone with a diameter of 25-mm filter (Sterlitech) was placed in filter cassettes to collect particulates for DNA and RNA extraction. An in-house-designed, 3D printed cartridge was placed at the end of the airflow, which contained 200 mg of zeolite adsorbent beads (Sigma-Aldrich 2-0304) to collect chemicals. Before deployment to the participant, the MicroPEM was calibrated to a flow rate of 0.5 L/min (±5%) using a mass flow meter (TSI 4140). During the study, the participant placed the exposometer on his arm or within a radius of 2 m. Samples were collected after 1–3 d of use (Fig. 1B) and stored at −80°C until analysis. To minimize the potential contamination, filters and related components were handled in sterile biological safety cabinets and cleaned with ethanol before use. Clean polyethersulfone filter and zeolite adsorbent beads were included before extraction as background controls. MicroPEM log files were downloaded using Docking Station software (RTI International). The participant used the MOVES app to track geographic locations through GPS coordinates and daily activities (Jiang et al. 2018). General environmental data were collected from the exposometer or National Oceanic and Atmospheric Administration's National Climatic Data Center or National Centers for Environmental Information.

### Analysis of chemical exposome by LC-HRMS

LC-HRMS was performed on a platform composed of a Waters UPLC coupled with Exactive Orbitrap mass spectrometer (Thermo Fisher Scientific) using a mixed-mode OPD2 HP-4B column (4.6 mm × 50 mm) with a guard column (4.6 mm × 10 mm; Shodex, Showa Denko). The column temperature was maintained at 45°C and the sample chamber at 4°C. The binary mobile phase solvents were as follows: A, 10 mM ammonium acetate in 50:50 (vol/vol) acetonitrile:water; B, 10 mM ammonium acetate in 90:10 (vol/vol) acetonitrile:water. Both solvents were modified with 10 mM acetic acid (pH 4.75) for positive mode acquisition or 10 mM ammonium acetate (pH 9.25) for negative mode. The flow rate was set as follows: flow rate, 0.3 mL/min; gradient—0–15 min, 99% A, 15–18 min, 99% to 1% A; 18–24 min, 1% A; 24–25 min, 1%–99% A; 25–30 min, 99% A. The MS acquisition was set as full scan mode with an electrospray ionization probe. The capillary temperature was 275°C; the sheath gas was 40 units; and the positive mode spray voltage was 3.5 kV, with 3.1 kV for the negative mode. The capillary voltage was 30 V; the tube lens voltage was 120 V; and the skimmer voltage was 20 V. The mass spectrum scan used 100,000 mass resolution, high dynamic range for the AGC target, a maximum injection time of 500 msec, and a scan range of 70–1000 m/z. The details of quality assurance and quality control of both targeted and nontargeted analyses were described in a previous study (Jiang et al. 2021). The raw and processed data used in this study were previously published and deposited to the NCBI BioProject database (https://www.ncbi.nlm.nih.gov/bioproject/) under accession number PRJNA421162. The details of all the data used in this study can be found in the supplemental data files of the previous publications as well (Jiang et al. 2018).

### Post-acquisition analysis of the chemical exposome

Analysis of chemical exposome was performed as previously described (Jiang et al. 2018, 2021). In brief, feature detection was performed with XCMS. For a conservative assessment of the number of unique chemical features, a customized Python script was used to remove potential isoforms, isotopes, and adducts from the 3299 features enriched at least 10-fold compared with the blank control (Jiang et al. 2018, 2021). The annotation was based on various exposome-related databases (blood exposome [https://bloodexposome.org/], T3DB [http://www.t3db.ca/], Exposome-Explorer [http://exposome-explorer.iarc.fr/], and HMDB [https://hmdb.ca/]) that are publicly available (Wishart et al. 2015; Barupal and Fiehn 2019; Neveu et al. 2020), as well as in-house databases by metID (Shen et al. 2022). The annotation confidence levels of all the chemicals in the exposome were at least level 5, with at least one chemical at level 1 in each chemical class, such as diethyltoluamide, diethylene glycol, decanoic acid, omethoate, octanoic acid, pyridine, phthalic acid, and hexanoic acid (Shen et al. 2019; Jiang et al. 2021). Because most of the exposome databases do not contain experimental MS^2^ data and this is a nontarget exposomics study, the filter of the confidence levels higher than three was not applied.

### Sequencing and analysis of biological exposome

DNA and RNA sequencing and analysis were performed as previously described (Jiang et al. 2018, 2021). In brief, DNA and RNA were extracted from filters and linearly amplified for sequencing. Libraries were sequenced by the Illumina HiSeq 4000 platform (2 × 151 bp), which yields an average of about 50 million unique reads per sample. Sequenced reads were deduplicated, and adapters were trimmed using Trim Galore! (version 0.4.4). Human-related reads were identified using BWA mapped to the GRCh37 human genome and removed. Although the GRCh37 human genome is not the latest version, the differences between GRCh37 and GRCh38 will not impact the results in this study because GRCh37 was only used to remove the human genome in the biological exposome. Specifically, we did not include the human genome in our taxonomy classification steps; therefore, even if the new version of GRCh38 human genome would include slightly more information, it will not affect our taxonomy classification process. Moreover, the biological annotation step was based on the microbiology databases, and the presented results in the biological exposome do not contain any human genome information. Following dehumanization, nonhuman reads were used for de novo assembly using MEGAHIT (Li et al. 2015) (1.1.1), and contigs were queried against our in-house database with BLASTN (2.3.9+) wrapper. The extensive in-house reference genome database included more than 40,000 species covering all domains of life (Jiang et al. 2018). Taxonomy classification and abundance were determined using a customized lowest common ancestor (LCA) algorithm (Jiang et al. 2018, 2021). The raw and processed sequencing data used in this study were previously published and deposited to the NCBI BioProject database (https://www.ncbi.nlm.nih.gov/bioproject/) under accession number PRJNA421162. The previous analysis script, the detailed information for contigs assigned as Rotifer and Apicomplexa, and the details of all of the data used in this study can be found in the supplemental data files of previous publications as well (Jiang et al. 2018).

### Blood sample collection

At the designated time point, blood was drawn from the overnight fasted participant in the Clinical and Translational Research Unit at Stanford University. Aliquots of blood were condensed at room temperature to coagulate, and clots were subsequently pelleted. The serum supernatant was then immediately frozen at −80°C. The blood in the EDTA tubes was immediately layered onto the Ficoll medium and spun with gradient centrifugation. Then the top layers were removed, and plasma was aliquoted and immediately frozen at −80°C. Subsequently, blood mononuclear cells (PBMCs) were collected and counted using a cell counter. Aliquots of PBMCs were further pelleted and frozen with DMSO/FBS. For the later multi-omics analyses, PBMCs were thawed on ice and then lysed to protein fraction using AllPrep spin columns (Qiagen) according to the manufacturer's instructions with the QIAshredder lysis option. Upon receipt of samples, blood samples were then stored at −80°C for clinical tests. The results of the blood and cytokines tests used in this study can be found in Supplemental Data S1 (Schüssler-Fiorenza Rose et al. 2019). All raw and processed clinical tests data used in this study were previously published and deposited to the NIH Integrative Human Microbiome Project (iHMP) site (https://portal.hmpdacc.org). The details of all of the data used in this study can be found in the supplemental data files of previous publications as well (Schüssler-Fiorenza Rose et al. 2019; Zhou et al. 2019).

### Collection and analysis of the gut microbiome

Stool samples were collected according to the Human Microbiome Project-Core Microbiome Sampling Protocol A (https://www.hmpdacc.org/). Following the Human Microbiome Project-Core Microbiome Sampling Protocol A (HMP Protocol 07-001, v12.0), DNA extraction was performed. We used the MOBIO PowerSoil DNA extraction kit and Proteinase K to isolate DNA in a clean fume hood. Samples were then treated with lysozyme and staphylococcal hemolysin. For 16S (bacterial) rRNA gene amplification, the primers 27F and 534R (27F: 5′-AGAGTTTGATCCTGGCTCAG-3′; 534R: 5′-ATTACCGCGGCTGCTGG-3′) were used to amplify the 16S hypervariable regions V1–V3. Unique barcode amplicons were used, and samples were sequenced on the Illumina MiSeq platform (V3; 2 × 300 bp). Illumina software handled the initial processing of all raw sequencing data. Reads were further processed by removing low-quality (average quality < 35) and ambiguous base (Ns) sequences. UCHIME (Edgar et al. 2011) was used to remove chimeric amplicons, cluster the amplicon sequences, and select the operational taxonomic unit by USEARCH (Edgar 2010) based on the Greengenes database (version in May 2013) (DeSantis et al. 2006). The final biological classification assignment was performed using the RDP-classifier in QIIME with custom scripts (Schüssler-Fiorenza Rose et al. 2019; Zhou et al. 2019). All raw and processed gut microbiome data used in this study were previously published and deposited to the iHMP site (https://portal.hmpdacc.org). The details of all of the data used in this study can be found in the supplemental data files of previous publications as well (Schüssler-Fiorenza Rose et al. 2019; Zhou et al. 2019).

### Untargeted proteomics by LC-HRMS

Preparation and analysis of plasma samples were performed as previously described (Zhou et al. 2019). In short, tryptic peptides from plasma samples were separated on the NanoLC 425 system (SCIEX); 0.5 × 10 mm ChromXP (SCIEX) was used for trap-elution settings, and the flow rate was set to 5 µL/min. The LC gradient was a 43-min gradient with mobile phase A, 0.1% formic acid in 100% water, and mobile phase B, 0.1% formic acid in 100% acetonitrile. During the gradient, mobile phase B was 4%–32%. Then 8 µg of undepleted plasma was loaded on LC. SWATH acquisition was performed on a TripleTOF 6600 system equipped with a DuoSpray source and a 25-µm ID electrode (SCIEX). The variable Q1 window SWATH acquisition mode (100 windows) was constructed in the high-sensitivity MS^2^ mode. PyProphet was used to score the peak groups in each run statistically, and TRIC was used to align all runs (Röst et al. 2016). Finally, a matrix with a peptide level of 1% FDR and a protein level of 10% FDR was generated for subsequent analysis. The protein abundances were the sum of the first three most abundant peptides. Perseus (v 1.4.2.40) was applied to subtract the main components showing the main batch deviation to reduce the batch effect (Zhou et al. 2019). All raw and processed proteome data used in this study were previously published and deposited to the iHMP site (https://portal.hmpdacc.org). The details of all of the data used in this study can be found in the supplemental data files of previous publications as well (Schüssler-Fiorenza Rose et al. 2019; Zhou et al. 2019).

### Cytokine profiling

The levels of circulating cytokines in the blood were measured by the 62-plex Luminex antibody-conjugated magnetic bead capture assay (Affymetrix), which has been extensively characterized and benchmarked by the Stanford Human Immunological Monitoring Center. The human 62-plex (eBiosciences/Affymetrix) was used with the modifications described below. Briefly, beads were added to a 96-well plate and washed using Biotek ELx405. Samples were added to the plate containing mixed antibody-linked beads and incubated for 1 h at room temperature and then overnight at 4°C with shaking (500–600 rpm, orbital shaker). After overnight incubation, the plate was washed, and then the biotinylated antibody was added. The plate was incubated for 75 min at room temperature with shaking. The plate was washed, and streptavidin-PE was added for detection. After incubating for 30 min at room temperature, the plate was washed once, and then the reading buffer was added to the wells. The plate was read by a Luminex 200 instrument, and the lower limit of each cytokine per sample was set to 50 beads. Radix Biosolutions custom assay control beads were added to all wells. The batch effect was corrected using replicates and controls shared between batches (Zhou et al. 2019). All raw and processed cytokines data used in this study were previously published and deposited to the iHMP site (https://portal.hmpdacc.org). The details of all of the data used in this study can be found in the supplemental data files of previous publications as well (Schüssler-Fiorenza Rose et al. 2019; Zhou et al. 2019).

### Untargeted metabolomics by LC–HRMS

All blood samples were prepared and analyzed for metabolomics as previously described (Contrepois et al. 2015). In short, plasma samples were extracted with acetone:acetonitrile:methanol (1:1:1 vol/vol/vol), evaporated to dryness under nitrogen, and reconstituted in methanol:water (1:1 vol/vol) for LC-HRMS analysis. HILIC and RPLC separations were used to analyze the extractants four times in positive and negative modes, respectively. HILIC metabolomics data were obtained on a Q Exactive plus, and RPLC metabolomics data were obtained on a Q Exactive (Thermo Fisher Scientific). Both instruments were equipped with HESI-II probes and operated in the full MS scan mode. We only used the combined quality control samples from the study to obtain MS^2^ data. We used a ZIC-HILIC column (2.1 × 100 mm, 3.5 µm, 200 Å; Merck Millipore) and mobile phases composed of 10 mM ammonium acetate in acetonitrile:water (50:50 vol/vol; A) and 10 mM ammonium acetate in acetonitrile/water (95:5 vol/vol; B), and a Zorbax SB-Aq column (2.1 × 50 mm, 1.7 µm, 100 Å; Agilent Technologies) and mobile phases composed of 0.06% acetic acid in water (A) and 0.06% acetic acid in methanol (B) to perform HILIC and RPLC analyses, respectively. All raw metabolomics data were processed using Progenesis QI (Nonlinear Dynamics, Waters). We also removed features that did not show sufficient linearity when diluted. Only features presented in more than one-third of samples were retained for further analysis, and the KNN method was used to estimate missing values. To normalize the data, locally assessed scatter plot smoothness analysis was applied (Dunn et al. 2011). Metabolic signatures were identified by matching retention time and fragmentation spectra to corresponding standards or comparing fragmentation patterns to public repositories, as previously reported (Zhou et al. 2019). Toxin and carcinogens were annotated out of the metabolome features if the feature could not be annotated as a human metabolite. The annotations of toxins and carcinogens were based on various blood-exposome-related databases that are publicly available as well as in-house databases (Wishart et al. 2015; Barupal and Fiehn 2019; Neveu et al. 2020). The confidence levels of metabolites annotations were at least level 3 based on our in-house databases (Shen et al. 2019), whereas blood toxins and carcinogens annotation were at least level 5 as a nontarget screening like the external chemical exposome owing to lack of experimental MS^2^ databases. All raw and processed metabolome data used in this study were previously published and deposited to the iHMP site (https://portal.hmpdacc.org). The details of all of the data used in this study can be found in the supplemental data files of previous publications as well (Schüssler-Fiorenza Rose et al. 2019; Zhou et al. 2019).

### General statistical analysis and data visualization

All statistical analysis and data visualization were performed using R (v3.6.0) (R Core Team 2021) and RStudio (v 1.2.5019) (RStudio Team 2021; https://www.rstudio.com). Most of the R packages and their dependencies used in this study were deployed in CRAN (https://cran.r-project.org) or Bioconductor (https://bioconductor.org/), and some of them are deployed on GitHub. Session information for this study is provided in Supplemental Code S1. All scripts to reproduce analyses and data visualization for this study are provided in Supplemental Code S2 and available on GitHub (https://github.com/jaspershen/precision_exposome/). All data from the exposome and internal -ome data were log_2_ transformed before analysis. Two kinds of fiber intakes were statistically adjusted for all internal -ome data to reduce fiber intake biases according to the participant's food log. Specifically, the participant took 20 g arabinoxylan daily from January 15, 2016 to January 31, 2016 and took 10 g guar gum daily from February 22, 2016 to March 17, 2016.

### Exposome and internal multi-omic correlation networks

Spearman's correlation was used to build the correlations in the intra/inter-omics analyses because it provided the most significant correlations as testable hypotheses. In general, for each two -omes pair, the correlation matrix was calculated as below; for each variable in one -ome, Spearman's correlations and FDR-adjusted *P*-values were generated with all features in the other -omes. Only correlations between each pair variable with absolute correlation > 0.9 and FDR-adjusted *P*-value < 0.05 were kept to construct the final correlation networks.

### Community analysis

Community analysis was performed based on edge betweenness embedded in the R package igraph (https://igraph.org/). Briefly, this is an iterative process, the edges with the highest edge betweenness score were removed in each iteration, and the process was repeated until only individual nodes remain. At each iteration, modularity was calculated, and communities were analyzed at the iteration that maximized this quantity. A visualization of iteration community versus modularity is shown in Supplemental Figure S6A and S6B. To ensure the robustness and reliability of our findings, only communities (or clusters) with at least three nodes were kept for subsequent analysis. All the networks were visualized using R package igraph, ggraph, and tidygraph.

### GO, KEGG, and Reactome pathway enrichment for proteome

The R package ClusterProfiler (v 3.18.0) was used for GO, KEGG, and Reactome pathway enrichment for proteomics. In general, UniProt and ENTREZID were obtained for proteins that connect with the exposome in correlation networks. Then the GO, KEGG, and Reactome pathway databases were used for pathway enrichment (hypergeometric distribution test, *P*-values are adjusted by the FDR method, and the cutoff was set as 0.05). Only pathways with a hitting protein number > 3 were retained for subsequent analysis.

### Metabolic feature–based dysregulated module detection

Applying the same concept from mummichog (Li et al. 2013) and PIUMet (Pirhaji et al. 2016), metabolic networks from KEGG and community analysis were used to detect dysregulated modules based on metabolic features connecting the exposome, respectively (Li et al. 2013). In general, the metabolic network (MN) was downloaded from KEGG, which contains 1377 nodes (metabolites) and 1561 edges (reactions). The brief workflow is described below:

1. All the metabolic features connecting the exposome (L_sig_) were matched with the KEGG metabolite database based on different adducts (Supplemental Table S1). Then all matched metabolites (significant metabolites) were mapped in the metabolic network to get the subnetwork (SN). Nonsignificant metabolites (hidden metabolites) that can connect significant metabolites within three reactions were also included in the subnetwork. Then the modules (M) were detected in the subnetwork via random walks (Yolum et al. 2005). Only modules with at least three nodes were kept. These modules were named significant modules (M_sig_) from real biological-related metabolic features.
2. For each module, the activity score (S) was calculated to measure both the modularity and enrichment of input metabolites (I). The activity score (S) of the module (M) was calculated as follows:

For a module M,

$$S=Q*\frac{N_{I,M}}{N_{M}},$$

where S is the activity score, *N*_*M*_ is the metabolite number in module *M*, and *N_I,M_* is the input metabolite number in module *M*. Q is the adjusted Newman–Girvan modularity calculated as below:

$$Q=\sqrt{\frac{N_{I}}{N_{M}}}*\left(\frac{E_{M}}{m}-\sum_{i,j}\frac{k_{i}}{2m}*\frac{k_{j}}{2m}\;\right),\;\;i,\;j\in M,$$

where *k*_*i*_ is the degree of metabolite i in module *M*, *m* is the total number of edges in the metabolic network MN, *E*_*M*_ is the total number of edges in module *M*, and *N*_*I*_ is the number of input metabolites. The original Newman–Girvan modularity has a bias toward larger modules, and $\sqrt{\frac{N_{I}}{N_{M}}}$ was used to reduce this bias.

3. Then the NULL metabolic features (L_null_, the same number with L_sig_) were selected from all metabolic features (exclude L_sig_), and then steps 1 and 2 were repeated 100 times to generate a list of NULL modules (M_null_) and their activity score (S_null_).
4. Using maximum likelihood estimation, S_null_ was modeled as a Gamma distribution, and a cumulative distribution function (CDF) was calculated. The *P*-value for each significant module was then calculated, and only modules with the *P*-value < 0.05 remained.

The annotation results from this method were also compared with the annotation results from the Untargeted Metabolomics by LC-HRMS section provided in Supplemental Data S10. These results showed that annotations from this method have high specificity.

### KEGG pathway enrichment analysis for metabolomics data

The KEGG pathway database was downloaded from KEGG (https://www.genome.jp/kegg/) using the R package KEGGREST. Pathway enrichment analysis was used in the hypergeometric distribution test; *P*-values were adjusted by the FDR method; and only pathways with FDR-adjusted *P*-value < 0.05 were kept.

### Exposome contributions to cytokine and blood test

To calculate the contributions of the exposome on each cytokine and blood test, principal components (PCs) were first extracted for each exposome component, and only PCs with a cumulative explained variation > 80% were kept. Then the linear regression model was constructed using each cytokine/blood test as *y* and corresponding exposome component's PCs as *x*. *R*^2^ was extracted and used to represent the contributions of the exposome to each cytokine/blood test. To calculate the contribution of the exposome components, partial least squares (PLS) and variable important projection (VIP) were calculated. Lastly, $R^{2}*\frac{VIP_{i}}{sum(VIP)}\;\;$ (i ∈ chemical, biological, and environment) was used to represent the contributions of the exposome components on cytokine/blood tests.

## Data access

The newly annotated exposome results in this study can be found in Supplemental Data S1. The raw and processed microbiome, proteome, metabolome, cytokines, and blood tests data that were newly annotated in this study were deposited in the Stanford iPOP database (http://hmp2-data.stanford.edu/; Subject#: ZOZOW1T) and provided in Supplemental Data S1 (Subject#: 69-001). All data used for reproductive analysis can be found on GitHub (https://github.com/jaspershen/precision_exposome) and were provided in Supplemental Data S1–S10.

## Supplementary Material

Supplemental Material
