# Supplementary material for: Precision environmental health monitoring by longitudinal exposome and multi-omics profiling
Source: Genome Res. 2022 Jun;32(6):1199–214. doi: 10.1101/gr.276521.121 (PMC9248886; doi:10.1101/gr.276521.121)
Supplement: Supplemental Material [file supp_32_6_1199__DC1.html]

Precision environmental health monitoring by longitudinal exposome and multi-omics profiling — Supplemental Material 

# Precision environmental health monitoring by longitudinal exposome and multi-omics profiling

## Supplemental Material

- Supplemental\_Fig\_S1.docx
- Supplemental\_Fig\_S2.docx
- Supplemental\_Fig\_S3.docx
- Supplemental\_Fig\_S4.docx
- Supplemental\_Fig\_S5\_.docx
- Supplemental\_Fig\_S6.docx
- Supplemental\_Fig\_S7.docx
- Supplemental\_Fig\_S8\_.docx
- Supplemental\_Table\_S1.docx
- Supplemental\_Code\_S1.docx
- Supplemental\_Code\_S2.docx
- Supplemental\_Data\_S1.xlsx
- Supplemental\_Data\_S2.xlsx
- Supplemental\_Data\_S3.xlsx
- Supplemental\_Data\_S4.xlsx
- Supplemental\_Data\_S5.xlsx
- Supplemental\_Data\_S6.xlsx
- Supplemental\_Data\_S7.xlsx
- Supplemental\_Data\_S8.xlsx
- Supplemental\_Data\_S9.xlsx
- Supplemental\_Data\_S10.xlsx
