# Supplementary material for: Precision environmental health monitoring by longitudinal exposome and multi-omics profiling
Source: Genome Res. 2022 Jun;32(6):1199–214. doi: 10.1101/gr.276521.121 (PMC9248886; doi:10.1101/gr.276521.121)
Supplement: Supplemental Material [file supp_gr.276521.121_Supplemental_Fig_S1.docx]

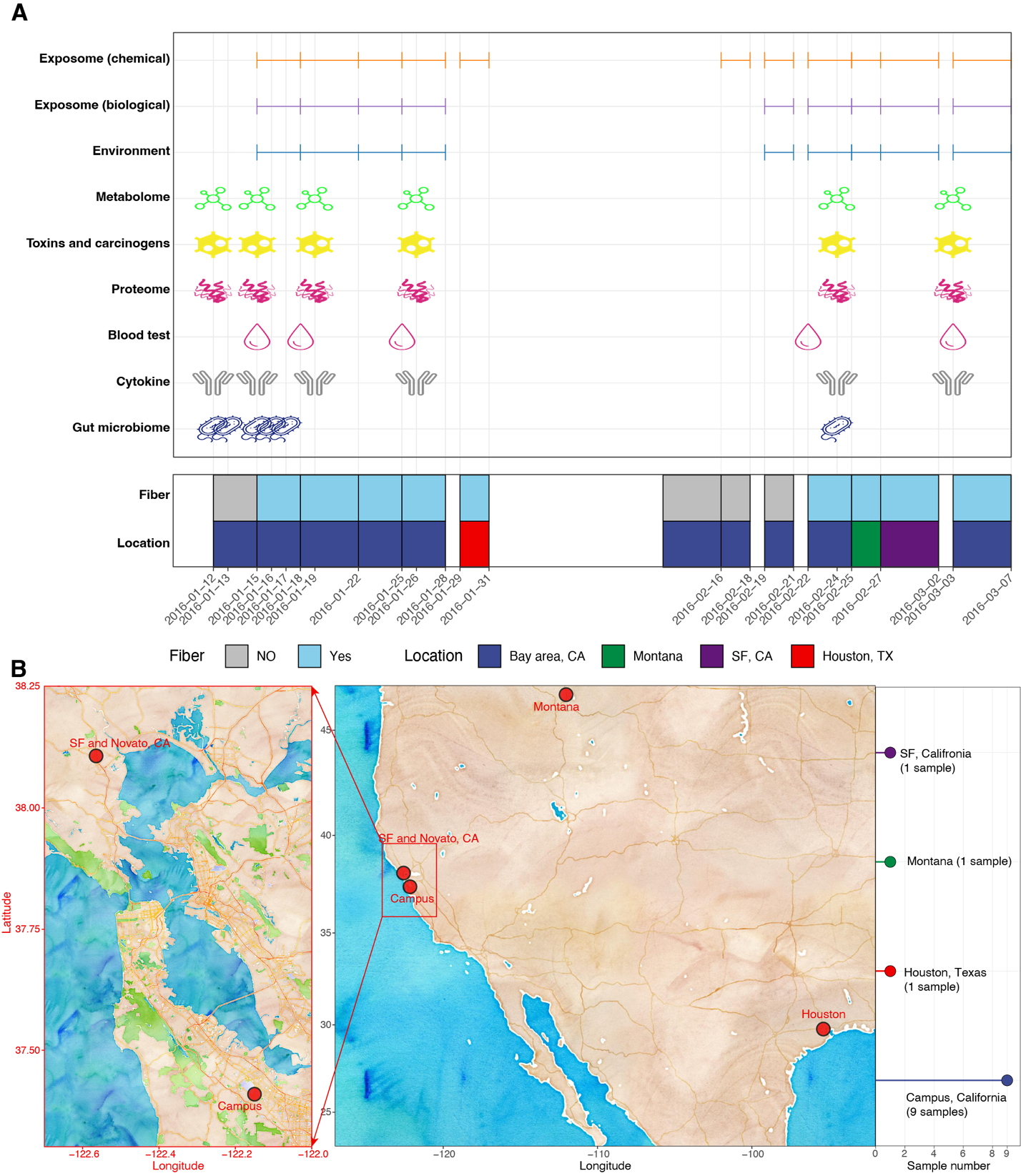


**Figure S1.** A detailed overview of sample collection. (a) Sample collection time points/periods for all exposome and internal multi-omics datasets. Corresponding fiber intake and geographical locations were also provided. (b) Collection locations for the exposome samples.
