# Supplementary material for: Precision environmental health monitoring by longitudinal exposome and multi-omics profiling
Source: Genome Res. 2022 Jun;32(6):1199–214. doi: 10.1101/gr.276521.121 (PMC9248886; doi:10.1101/gr.276521.121)
Supplement: Supplemental Material [file supp_gr.276521.121_Supplemental_Fig_S2.docx]

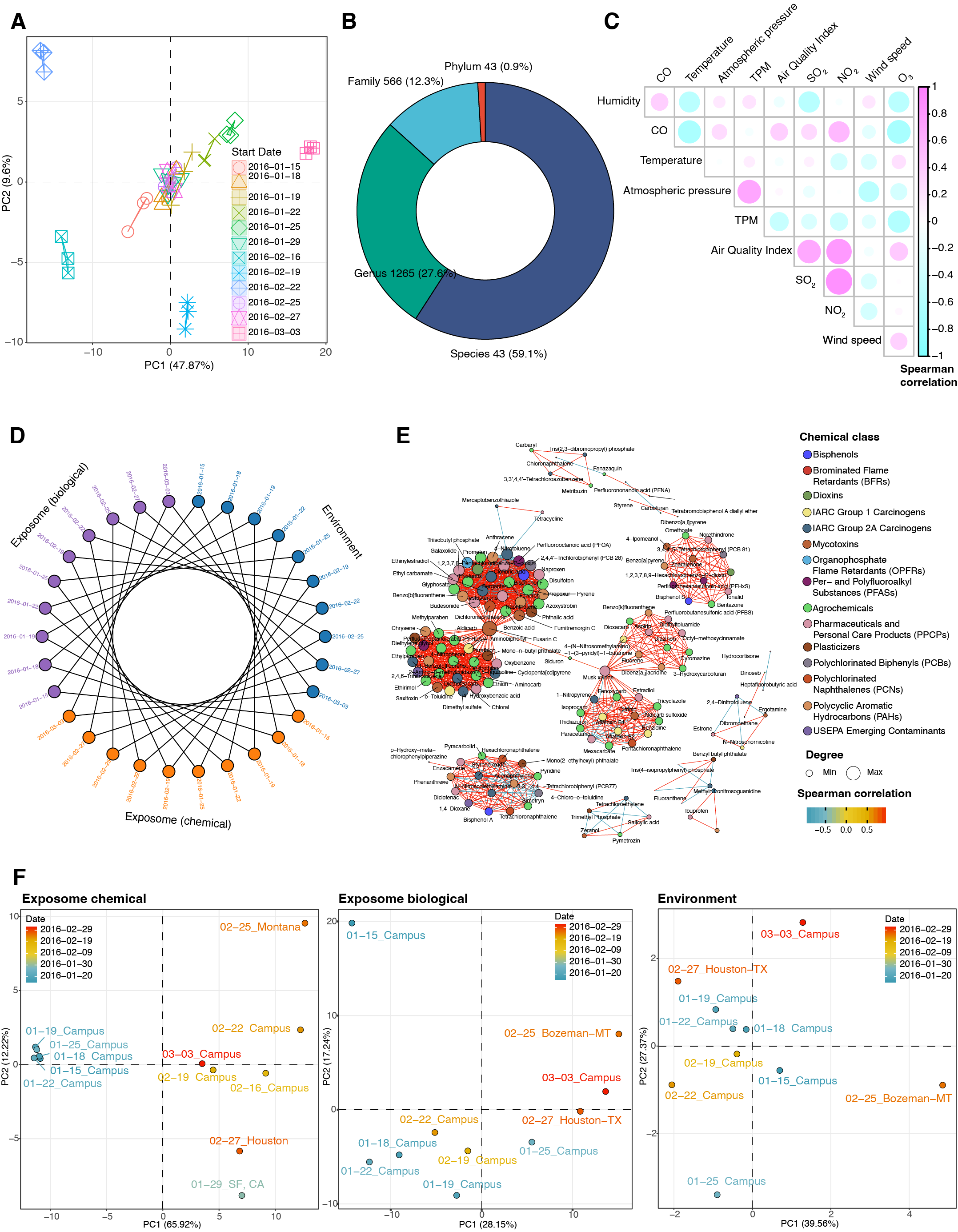


**Figure S2.** Overview of the exposome data. (a) PCA plot shows the data quality of chemical exposome data. For each sample, 3 repeats were acquired. (b) The number and percentage of biological exposome data at different taxonomic ranks. (c) Correlation plots with coefficients among all environmental factors. (d) Sample matching for 3 exposome domains. (e) Intra correlation network for the chemical exposome. (f) PCA plots of the 3 exposome domains based on the sampling time and locations.
