# Supplementary material for: Precision environmental health monitoring by longitudinal exposome and multi-omics profiling
Source: Genome Res. 2022 Jun;32(6):1199–214. doi: 10.1101/gr.276521.121 (PMC9248886; doi:10.1101/gr.276521.121)
Supplement: Supplemental Material [file supp_gr.276521.121_Supplemental_Fig_S3.docx]

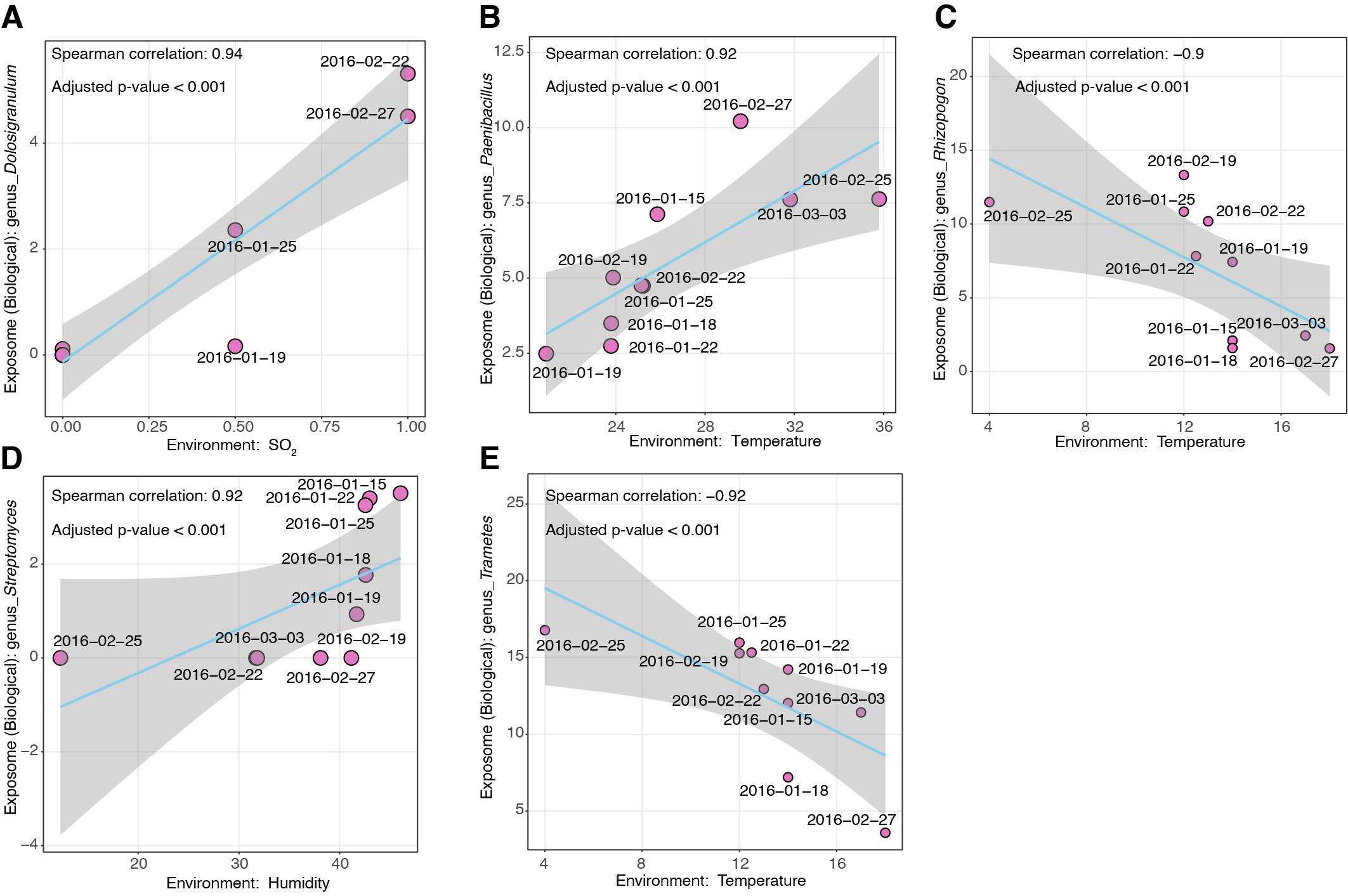


**Figure S3.** Representative Spearman correlation plots between environment factors and microbes.
