# Supplementary material for: Precision environmental health monitoring by longitudinal exposome and multi-omics profiling
Source: Genome Res. 2022 Jun;32(6):1199–214. doi: 10.1101/gr.276521.121 (PMC9248886; doi:10.1101/gr.276521.121)
Supplement: Supplemental Material [file supp_gr.276521.121_Supplemental_Fig_S4.docx]

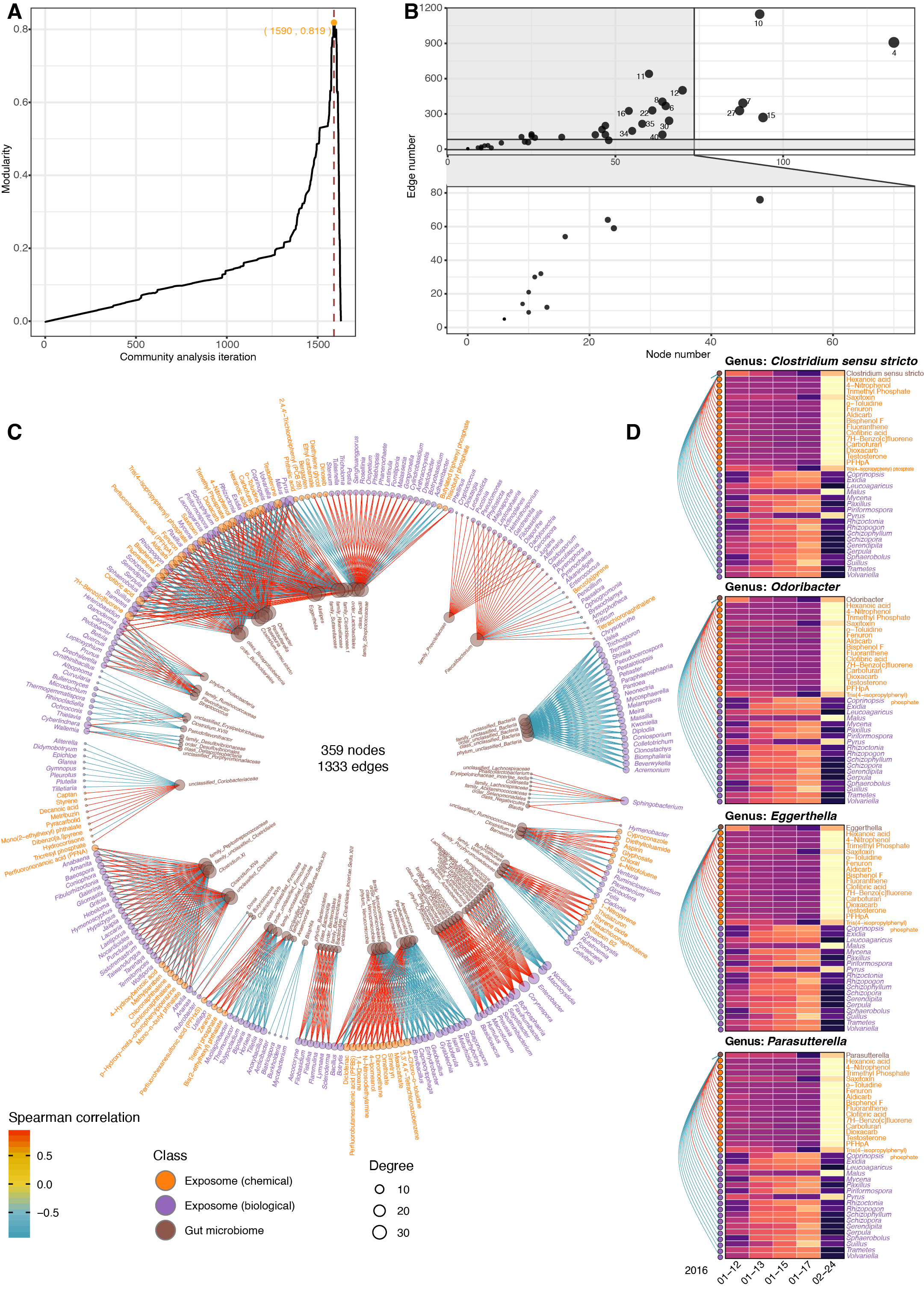


**Figure S4.** The exposome and internal multi-omics analyses. (a) The maximum modularity in the exposome and internal-omes correlation network community analysis was 0.819. (b) Node and edge numbers for all the subnetworks in the community analysis. (c) The complete correlation network between the exposome and the gut microbiome (|r| > 0.9; q-value < 0.05). (d) Representative personal gut bacteria that has 34 significant correlations with the exposome components (|r| > 0.9; q-value < 0.05).
