# Supplementary figures and images for: Precision environmental health monitoring by longitudinal exposome and multi-omics profiling
Source: Genome Res. 2022 Jun;32(6):1199–214. doi: 10.1101/gr.276521.121 (PMC9248886; doi:10.1101/gr.276521.121)

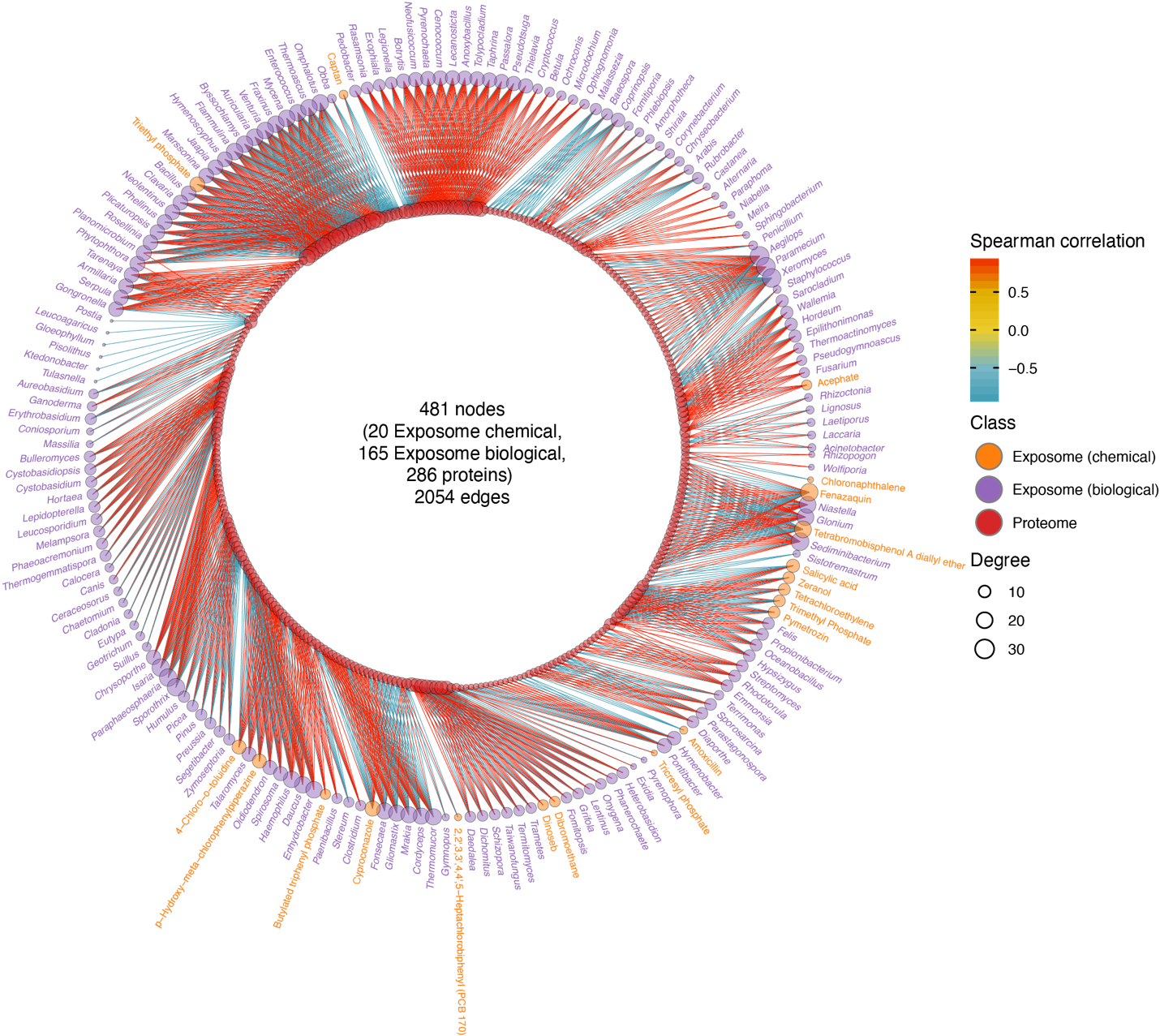


**Figure S5.** The complete correlation network between the exposome and proteome (|r| > 0.9; q-value < 0.05).

Supplement: Supplemental Material [file supp_gr.276521.121_Supplemental_Fig_S5_.docx]
