# Supplementary material for: Precision environmental health monitoring by longitudinal exposome and multi-omics profiling
Source: Genome Res. 2022 Jun;32(6):1199–214. doi: 10.1101/gr.276521.121 (PMC9248886; doi:10.1101/gr.276521.121)
Supplement: Supplemental Material [file supp_gr.276521.121_Supplemental_Fig_S6.docx]

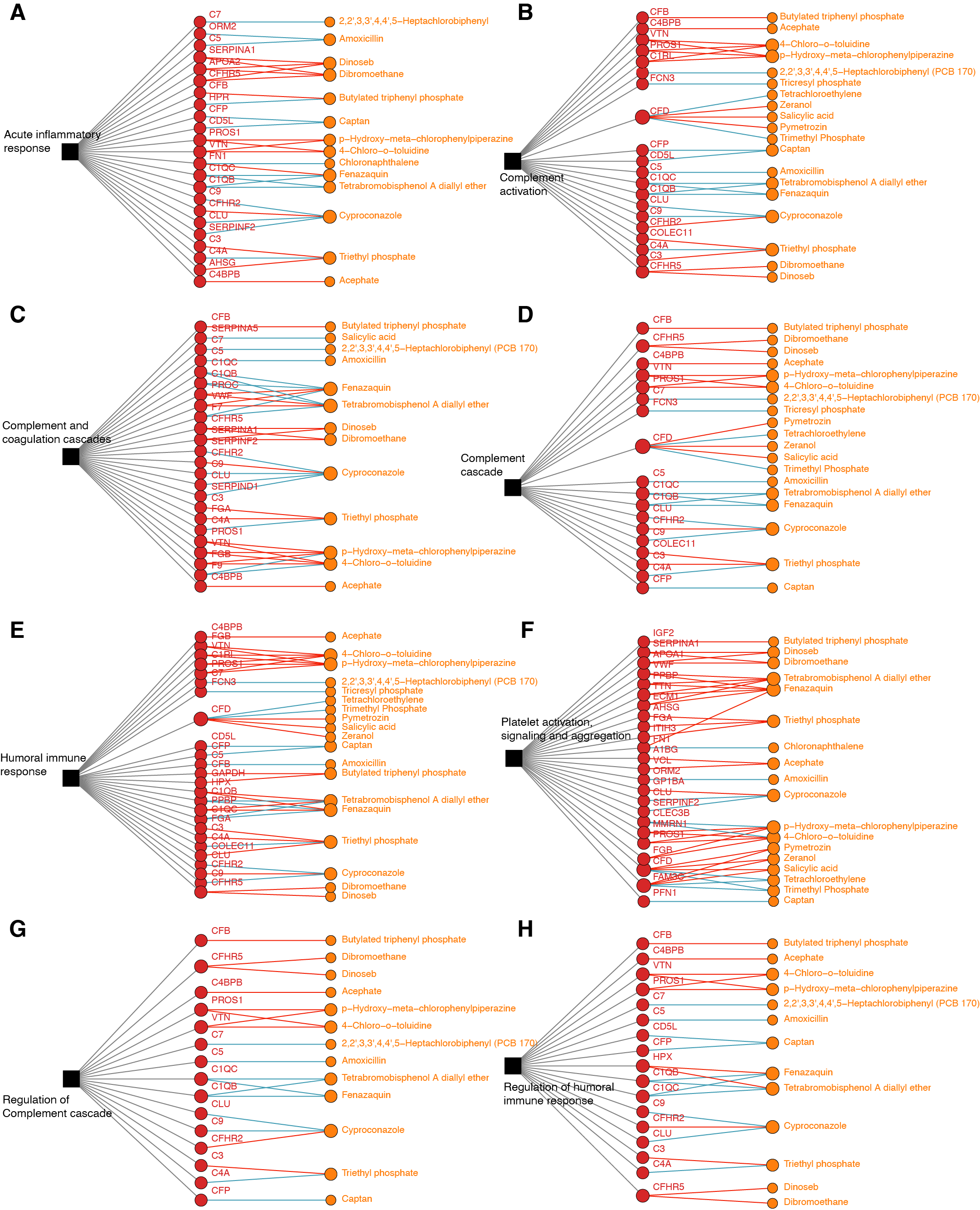


**Figure S6.** The detailed networks between the exposome and proteins for each signaling pathway (|r| > 0.9; q-value < 0.05).
