# Supplementary material for: Precision environmental health monitoring by longitudinal exposome and multi-omics profiling
Source: Genome Res. 2022 Jun;32(6):1199–214. doi: 10.1101/gr.276521.121 (PMC9248886; doi:10.1101/gr.276521.121)
Supplement: Supplemental Material [file supp_gr.276521.121_Supplemental_Fig_S7.docx]

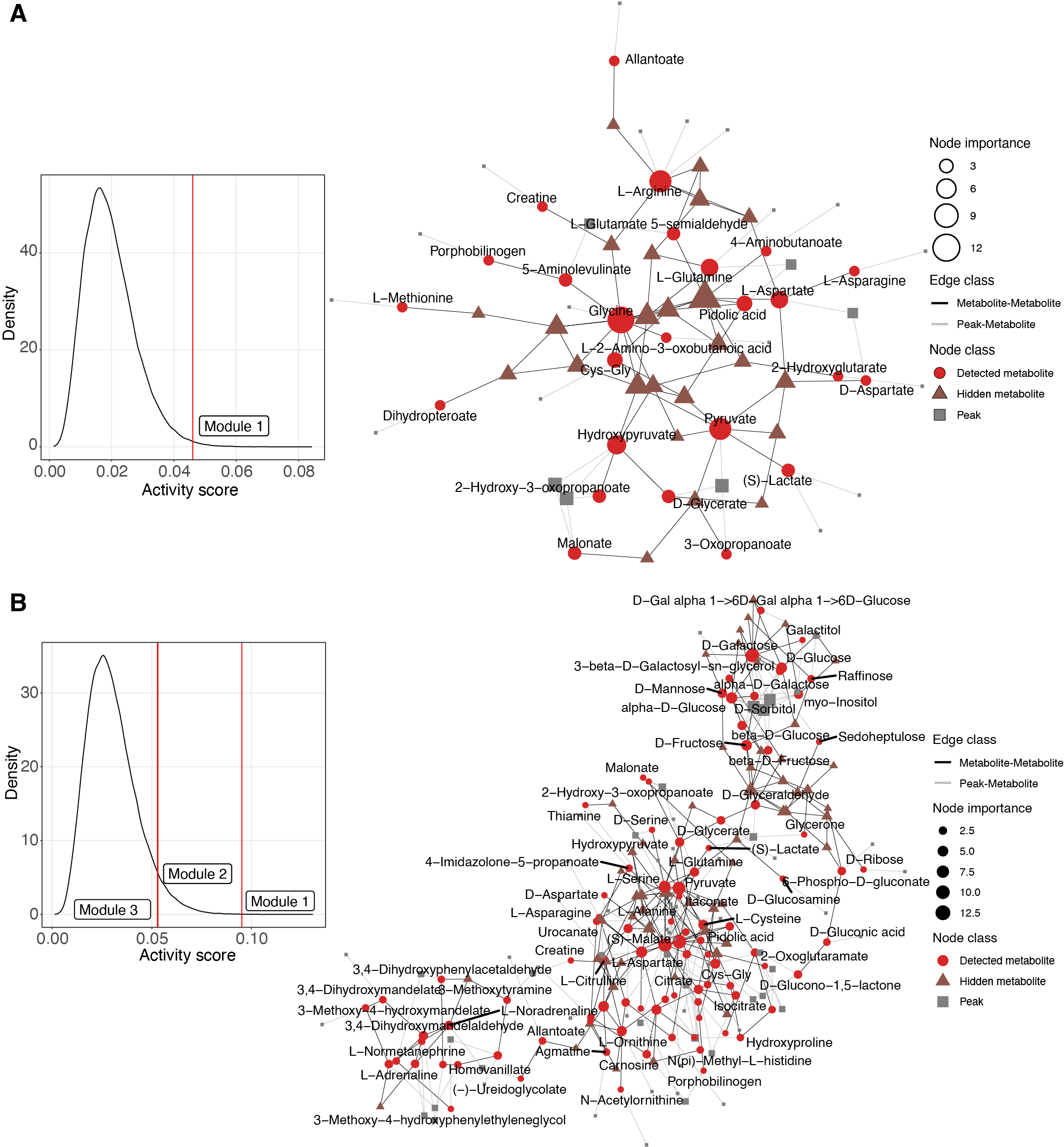


**Figure S7.** Feature-based network analysis for metabolic features significantly correlated with the exposome (**Methods**). (a) Chemical exposome. Left panel, the null distribution of activity scores and only module 1 was significant. Right panel, network constructed by significant modules. (b) Biological exposome. Left panel, the null distribution of activity scores and modules 1, 2 and 3 were significant. Right panel, network constructed by significant modules.
