# Supplementary material for: Precision environmental health monitoring by longitudinal exposome and multi-omics profiling
Source: Genome Res. 2022 Jun;32(6):1199–214. doi: 10.1101/gr.276521.121 (PMC9248886; doi:10.1101/gr.276521.121)
Supplement: Supplemental Material [file supp_gr.276521.121_Supplemental_Table_S1.docx]

**Supplemental Table S1. Adducts list for metabolites annotation in this study.**

| **LC-HRMS Mode** | **Adduct** |
| --- | --- |
| RPLC positive mode | (M+H)^+^, (M+H-H_2_O)^+^, (M+H-2H_2_O)^+^, (M+NH_4_)^+^, (M+Na)^+^, (M-H+2Na)^+^, (M-2H+3Na)^+^, (M+K)^+^, (M-H+2K)^+^, (M-2H+3K)^+^, (M+CH_3_CN+H)^+^, (M+CH_3_CN+Na)^+^, (2M+H)^+^, (2M+NH_4_)^+^, (2M+Na)^+^, (2M+K)^+^, (M+HCOO+2H)^+^ |
| RPLC negative mode | (M-H)^-^, (M-H_2_O-H)^-^, (M+Na-2H)^-^, (M+K-2H)^-^, (M+NH_4_-2H)^-^, (2M-H)^-^, (M+F)^-^ |
| HILIC positive mode | (M+H)^+^, (M+H-H_2_O)^+^, (M+H-2H_2_O)^+^, (M+NH_4_)^+^, (M+Na)^+^, (M-H+2Na)^+^, (M-2H+3Na)^+^, (M+K)^+^, (M-H+2K)^+^, (M-2H+3K)^+^, (M+CH_3_CN+H)^+^, (M+CH_3_CN+Na)^+^, (2M+H)^+^, (2M+NH_4_)^+^, (2M+Na)^+^, (2M+K)^+^, (M+HCOO+2H)^+^ |
| HILIC negative mode | (M-H)^-^, (M-H_2_O-H)^-^, (M+Na-2H)^-^, (M+K-2H)^-^, (M+NH_4_-2H)^-^, (2M-H)^-^, (M+CH_3_COO)^-^ |
