# Supplementary material for: Precision environmental health monitoring by longitudinal exposome and multi-omics profiling
Source: Genome Res. 2022 Jun;32(6):1199–214. doi: 10.1101/gr.276521.121 (PMC9248886; doi:10.1101/gr.276521.121)
Supplement: Supplemental Material [file supp_gr.276521.121_Supplemental_Code_S1.docx]

**Supplemental Code S1. The session information in the study.**

R version 4.0.3 (2020-10-10)

Platform: x86_64-apple-darwin17.0 (64-bit)

Running under: macOS Big Sur 10.16

Matrix products: default

LAPACK: /Library/Frameworks/R.framework/Versions/4.0/Resources/lib/libRlapack.dylib

locale:

[1] en_US.UTF-8/en_US.UTF-8/en_US.UTF-8/C/en_US.UTF-8/en_US.UTF-8

attached base packages:

[1] parallel stats4 grid stats graphics grDevices utils datasets methods

[10] base

other attached packages:

[1] ReactomePA_1.34.0 org.Hs.eg.db_3.10.0 AnnotationDbi_1.52.0 IRanges_2.24.1

[5] S4Vectors_0.28.1 Biobase_2.50.0 BiocGenerics_0.36.0 clusterProfiler_3.18.0

[9] circlize_0.4.11 ComplexHeatmap_2.6.2 patchwork_1.1.1 plsVarSel_0.9.6

[13] pls_2.7-3 openxlsx_4.2.3 plyr_1.8.6 ggalluvial_0.12.3

[17] tidygraph_1.2.0 ggraph_2.0.4 igraph_1.2.6 forcats_0.5.0

[21] stringr_1.4.0 dplyr_1.0.2 purrr_0.3.4 readr_1.4.0

[25] tidyr_1.1.2 tibble_3.0.4 ggplot2_3.3.3 tidyverse_1.3.0

loaded via a namespace (and not attached):

[1] readxl_1.3.1 shadowtext_0.0.7 backports_1.2.1

[4] fastmatch_1.1-0 sxtTools_0.99.01 splines_4.0.3

[7] BiocParallel_1.24.1 crosstalk_1.1.0.1 digest_0.6.27

[10] htmltools_0.5.0 GOSemSim_2.16.1 rsconnect_0.8.16

[13] magick_2.5.2 viridis_0.5.1 GO.db_3.10.0

[16] wesanderson_0.3.6 fansi_0.4.1 magrittr_2.0.1

[19] checkmate_2.0.0 memoise_1.1.0 cluster_2.1.0

[22] graphlayouts_0.7.1 modelr_0.1.8 matrixStats_0.57.0

[25] bdsmatrix_1.3-4 prettyunits_1.1.1 enrichplot_1.10.1

[28] colorspace_2.0-0 blob_1.2.1 rvest_0.3.6

[31] rappdirs_0.3.1 ggrepel_0.9.0 xfun_0.19

[34] haven_2.3.1 crayon_1.3.4 jsonlite_1.7.2

[37] graph_1.68.0 scatterpie_0.1.5 glue_1.4.2

[40] polyclip_1.10-0 gtable_0.3.0 webshot_0.5.2

[43] genalg_0.2.0 GetoptLong_1.0.5 graphite_1.36.0

[46] shape_1.4.5 scales_1.1.1 DOSE_3.16.0

[49] mvtnorm_1.1-1 DBI_1.1.0 miniUI_0.1.1.1

[52] Rcpp_1.0.5.4 progress_1.2.2 xtable_1.8-4

[55] viridisLite_0.3.0 clue_0.3-58 gridGraphics_0.5-1

[58] bit_4.0.4 reactome.db_1.70.0 htmlwidgets_1.5.3

[61] httr_1.4.2 fgsea_1.16.0 RColorBrewer_1.1-2

[64] ellipsis_0.3.1 pkgconfig_2.0.3 farver_2.0.3

[67] dbplyr_2.0.0 utf8_1.1.4 manipulateWidget_0.10.1

[70] later_1.1.0.1 ggplotify_0.0.5 tidyselect_1.1.0

[73] labeling_0.4.2 rlang_0.4.10 reshape2_1.4.4

[76] munsell_0.5.0 cellranger_1.1.0 tools_4.0.3

[79] downloader_0.4 cli_2.2.0 generics_0.1.0

[82] RSQLite_2.2.1 broom_0.7.3 evaluate_0.14

[85] fastmap_1.0.1 yaml_2.2.1 knitr_1.30

[88] bit64_4.0.5 fs_1.5.0 zip_2.1.1

[91] rgl_0.103.5 mime_0.9 praznik_8.0.0

[94] DO.db_2.9 xml2_1.3.2 compiler_4.0.3

[97] rstudioapi_0.13 MSQC_1.0.2 png_0.1-7

[100] reprex_0.3.0 tweenr_1.0.1 stringi_1.5.3

[103] lattice_0.20-41 Matrix_1.2-18 ggsci_2.9

[106] vctrs_0.3.6 pillar_1.4.7 lifecycle_0.2.0

[109] BiocManager_1.30.10 GlobalOptions_0.1.2 data. table_1.13.6

[112] cowplot_1.1.1 httpuv_1.5.4 qvalue_2.22.0

[115] R6_2.5.0 promises_1.1.1 gridExtra_2.3

[118] sessioninfo_1.1.1 MASS_7.3-53 assertthat_0.2.1

[121] rjson_0.2.20 withr_2.3.0 hms_0.5.3

[124] rmarkdown_2.6 rvcheck_0.1.8 Cairo_1.5-12.2

[127] ggnewscale_0.4.4 ggforce_0.3.2 shiny_1.5.0

[130] lubridate_1.7.9.2
