# Supplementary material for: Precision environmental health monitoring by longitudinal exposome and multi-omics profiling
Source: Genome Res. 2022 Jun;32(6):1199–214. doi: 10.1101/gr.276521.121 (PMC9248886; doi:10.1101/gr.276521.121)
Supplement: Supplemental Material [file supp_gr.276521.121_Supplemental_Code_S2.docx]

**Supplemental Code S2. All scripts to reproduce analyses and data visualization.**

##avoid source

no_function()

##load data

sxtTools::setwd_project()

library(tidyverse)

rm(list = ls())

setwd("data_20200511/environment/")

load("expression_data")

load("sample_info")

load("variable_info")

temp_data <-

expression_data

colnames(temp_data) <-

sample_info$STARTING_DATE %>%

stringr::str_replace("2016-", "")

rownames(temp_data) <- variable_info$true_name

temp_data <-

temp_data %>%

tibble::rownames_to_column(var = "variable_id") %>%

tidyr::pivot_longer(cols = -variable_id,

names_to = "date", values_to = "value")

temp_data$variable_id <-

factor(temp_data$variable_id, levels = c(

"Temperature", "Humidity", "Atmospheric pressure", "Wind speed",

"SO2", "NO2", "O3", "CO",

"Air Quality Index", "TPM"

))

plot <-

temp_data %>%

ggplot(aes(date, value)) +

# geom_rect() +

geom_line(aes(x = date, y = value, group = 1)) +

geom_point(shape = 21,

size = 5,

aes(fill = variable_id), show.legend = FALSE) +

scale_fill_manual(values = c(

"Temperature" = ggsci::pal_d3()(n = 10)[1],

"Humidity" = ggsci::pal_d3()(n = 10)[1],

"Atmospheric pressure" = ggsci::pal_d3()(n = 10)[1],

"Wind speed" = ggsci::pal_d3()(n = 10)[1],

"SO2" = ggsci::pal_d3()(n = 10)[3],

"NO2"= ggsci::pal_d3()(n = 10)[3],

"O3"= ggsci::pal_d3()(n = 10)[3],

"CO"= ggsci::pal_d3()(n = 10)[3],

"Air Quality Index"= ggsci::pal_d3()(n = 10)[3],

"TPM"= ggsci::pal_d3()(n = 10)[3]

)) +

labs(x = "", y = "") +

theme_bw() +

theme(

panel.grid = element_blank(),

axis.text.x = element_text(

angle = 45,

size = 12,

hjust = 1,

vjust = 1

),

axis.text.y = element_text(size = 10),

axis.title = element_text(size = 10)

) +

facet_wrap(facets = vars(variable_id), scales = "free_y", nrow = 3)

plot

# ggsave(plot, filename = "all_plot.pdf", width = 16, height = 8)

# for (i in 1:nrow(variable_info)) {

# cat(i, " ")

# temp_data <-

# data.frame(value = as.numeric(expression_data[variable_info$variable_id[i], ]),

# sample_info,

# stringsAsFactors = FALSE)

#

# plot <-

# temp_data %>%

# dplyr::mutate(STARTING_DATE = stringr::str_replace(STARTING_DATE, "2016-", "")) %>%

# ggplot(aes(x = STARTING_DATE, y = value)) +

# geom_point(shape = 21,

# size = 6,

# fill = ggsci::pal_d3()(n = 10)[1]) +

# ggrepel::geom_text_repel(aes(label = location)) +

# theme_bw() +

# labs(x = "", y = variable_info$variable_id[i]) +

# theme(

# panel.grid.minor = element_blank(),

# axis.text.x = element_text(

# angle = 45,

# size = 12,

# hjust = 1,

# vjust = 1

# ),

# axis.text.y = element_text(size = 12),

# axis.title = element_text(size = 13)

# )

#

# name1 <- paste(variable_info$variable_id[i], "_1.pdf", sep = "")

# name2 <- paste(variable_info$variable_id[i], "_2.pdf", sep = "")

# ggsave(plot,

# filename = name1,

# width = 7,

# height = 7)

# ggsave(plot,

# filename = name2,

# width = 14,

# height = 7)

# }

###PCA analysis

temp_expression_data <-

expression_data

temp_expression_data <-

temp_expression_data %>%

apply(1, function(x){

x[is.na(x)] <- min(x, na.rm = TRUE)

x

}) %>%

t()

colnames(temp_expression_data) <-

as.character(sample_info$STARTING_DATE)

#PCA analysis

###PCA for date

pca_object <-

prcomp(x = t(temp_expression_data), center = TRUE, scale. = TRUE)

library(wesanderson)

names(wes_palettes)

wes_palette(name = "Zissou1", n = 100, type = "continuous")

pal <-

wesanderson::wes_palette(name = "Zissou1", n = 100, type = "continuous")

as.Date_origin <- function(x) {

as.Date(x, origin = '1970-01-01')

}

plot <-

pca_object$x %>%

as.data.frame() %>%

rownames_to_column(var = "date") %>%

left_join(sample_info %>% dplyr::mutate(date = as.character(STARTING_DATE)),

by = c("date")) %>%

mutate(date2 = as.character(date)) %>%

mutate(date = as.integer(as.Date(date))) %>%

ggplot(aes(PC1, PC2, colour = date)) +

geom_vline(xintercept = 0,

linetype = 2,

color = "black") +

geom_hline(yintercept = 0,

linetype = 2,

color = "black") +

geom_point(shape = 21,

size = 4,

aes(fill = date),

color = "black") +

guides(colour = guide_colourbar(title = "Date")) +

scale_colour_gradientn(colours = pal,

labels = as.Date_origin) +

scale_fill_gradientn(colours = pal,

labels = as.Date_origin) +

ggrepel::geom_text_repel(aes(PC1, PC2, label = paste(date2, location, sep = "_")),

show.legend = FALSE) +

theme_bw() +

theme(

axis.text = element_text(size = 12),

axis.title = element_text(size = 13),

panel.grid.minor = element_blank(),

legend.position = c(1, 1),

legend.justification = c(1, 1),

legend.background = element_rect(fill = "transparent"),

plot.background = element_rect(fill = "transparent"),

panel.background = element_rect(fill = "transparent")

) +

labs(

x = paste("PC1 (", round(summary(pca_object)$importance[2, 1], 4) * 100, "%)", sep = ""),

y = paste("PC2 (", round(summary(pca_object)$importance[2, 2], 4) *

100, "%)", sep = "")

)

plot

# ggsave(plot,

# file = "pca_date.pdf",

# width = 7,

# height = 7)

###coorplot of all the environment

library(corrplot)

library(corrr)

col3 <- colorRampPalette(c("red", "white", "blue"))

temp_data = corrr::correlate(t(expression_data), method = "spearman", diagonal = 1) %>%

tibble::column_to_rownames(var = "term")

colnames(temp_data) =

rownames(temp_data) =

variable_info$true_name

plot =

corrplot(

corr = as.matrix(temp_data),

is.corr = TRUE,

type = "upper",

order = "hclust",

col = cm.colors(100),

diag = FALSE

)

temp_data

##avoid source

no_function()

##

sxtTools::setwd_project()

library(tidyverse)

load("data_20200511/microbiome/dna_sample_info")

dna_sample_info <- dna_sample_info[,1:16]

sxtTools::setwd_project()

setwd("data_20200511/environment/")

sample_info <- readxl::read_xlsx("Exposome_select_peng_metadata-DNA.xlsx")

expression_data <-

sample_info[, c(

"STARTING_DATE",

"temperature",

"humid",

"X_Mean_Sea_Level_PressurehPa",

"X_Mean_Wind_SpeedKm.h",

"Overall.AQI.Value",

"dSO2",

"dNO2",

"dOzone",

"dCO",

"total.particle"

)] %>%

as.data.frame() %>%

dplyr::filter(!is.na(STARTING_DATE))

rownames(expression_data) <- as.character(expression_data$STARTING_DATE)

expression_data <-

expression_data %>%

dplyr::select(-STARTING_DATE)

variable_info <-

sample_info[1:2, -1] %>%

t() %>%

as.data.frame() %>%

tibble::rownames_to_column(var = "variable_id") %>%

dplyr::filter(variable_id %in% colnames(expression_data))

colnames(variable_info)[2:3] = c("true_name", "unit")

sample_info <-

sample_info %>%

dplyr::select(STARTING_DATE) %>%

dplyr::filter(!is.na(STARTING_DATE)) %>%

dplyr::mutate(sample_id = as.character(STARTING_DATE)) %>%

dplyr::select(sample_id, everything())

expression_data <-

t(expression_data) %>%

as.data.frame()

expression_data <-

expression_data %>%

apply(2, function(x){

x <- as.numeric(x)

}) %>%

as.data.frame()

rownames(expression_data) <- variable_info$variable_id

sum(is.na(expression_data))

expression_data

# expression_data <-

# impute::impute.knn(data = as.matrix(expression_data))$data %>%

# as.data.frame()

sample_info$sample_id == dna_sample_info$date.start

sample_info <-

cbind(sample_info,

dna_sample_info[,-1])

sample_info$location[sample_info$location == "Mike_background"] <- "Campus"

library(openxlsx)

wb = createWorkbook()

modifyBaseFont(wb, fontSize = 12, fontName = "Arial Narrow")

addWorksheet(wb, sheetName = "Sample information", gridLines = TRUE)

addWorksheet(wb, sheetName = "Variable information", gridLines = TRUE)

addWorksheet(wb, sheetName = "Expression data", gridLines = TRUE)

freezePane(wb, sheet = 1, firstRow = TRUE, firstCol = TRUE)

freezePane(wb, sheet = 2, firstRow = TRUE, firstCol = TRUE)

freezePane(wb, sheet = 3, firstRow = TRUE, firstCol = FALSE)

writeDataTable(wb, sheet = 1, x = sample_info,

colNames = TRUE, rowNames = FALSE)

writeDataTable(wb, sheet = 2, x = variable_info,

colNames = TRUE, rowNames = FALSE)

writeDataTable(wb, sheet = 3, x = expression_data,

colNames = TRUE, rowNames = FALSE)

saveWorkbook(wb, "environment_data.xlsx", overwrite = TRUE)

save(expression_data, file = "expression_data")

save(sample_info, file = "sample_info")

save(variable_info, file = "variable_info")

##avoid source

no_function()

##load data

sxtTools::setwd_project()

library(tidyverse)

rm(list = ls())

load("data_20200511/gut_microbiome/variable_info")

gutmicrobiome_variable_info <- variable_info

setwd("data_analysis/environment_gutmicrobiome/")

load("gutmicrobiome_expression_data")

load("environment_expression_data")

load("gutmicrobiome_sample_info")

load("environment_sample_info")

load("environment_variable_info")

gutmicrobiome_sample_info$sample_id == colnames(gutmicrobiome_expression_data)

colnames(gutmicrobiome_expression_data) <-

as.character(gutmicrobiome_sample_info$CollectionDate)

colnames(environment_expression_data) <-

colnames(gutmicrobiome_expression_data)

dim(environment_expression_data)

#######correlation analysis

dim(environment_expression_data)

dim(gutmicrobiome_expression_data)

##correct fiber for gutmicrobime

gutmicrobiome_expression_data1 <-

purrr::map(

as.data.frame(t(gutmicrobiome_expression_data)),

.f = function(x) {

temp_data <-

data.frame(fiber = c(0,

0,

0,

1,

1),

x,

stringsAsFactors = FALSE)

lm_result <- lm(formula = x ~ fiber, data = temp_data)

lm_result$residuals

}

) %>%

do.call(rbind, .) %>%

as.data.frame()

temp_data <-

apply(gutmicrobiome_expression_data1, 1, function(x) {

(x - mean(x)) / sd(x)

}) %>%

t()

colnames(temp_data) <-

colnames(gutmicrobiome_expression_data1) <-

colnames(gutmicrobiome_expression_data)

##heatmap of gutmicrobiome

library(circlize)

col_fun = colorRamp2(

breaks = seq(min(temp_data), max(temp_data), length.out = 90),

colors =

viridis::magma(n = 100)[-c(1:10)],

transparency = 0

)

plot <-

temp_data %>%

ComplexHeatmap::Heatmap(

cluster_columns = FALSE,

show_column_names = TRUE,

show_row_names = FALSE,

clustering_method_rows = "ward.D",

clustering_method_columns = "ward.D",

clustering_distance_columns = "euclidean",

clustering_distance_rows = "euclidean",

col = col_fun,

km = 2,

border = TRUE,

row_dend_reorder = TRUE,

column_dend_reorder = TRUE,

column_names_rot = 45,

name = "Z-score"

)

plot <- ggplotify::as.ggplot(plot)

plot

ggsave(plot,

filename = "gutmicrobiome_plot/gut_microbiome_heatmap.pdf",

width = 7,

height = 7)

###heatmap of environment

plot <-

environment_expression_data %>%

apply(1, function(x) {

(x - mean(x)) / sd(x)

}) %>%

t() %>%

ComplexHeatmap::Heatmap(

cluster_columns = FALSE,

show_column_names = TRUE,

show_row_names = FALSE,

clustering_method_rows = "ward.D",

clustering_method_columns = "ward.D",

clustering_distance_columns = "euclidean",

clustering_distance_rows = "euclidean",

col = col_fun,

km = 2,

border = TRUE,

row_dend_reorder = TRUE,

column_dend_reorder = TRUE,

column_names_rot = 45,

name = "Z-score"

)

plot <- ggplotify::as.ggplot(plot)

plot

ggsave(plot,

filename = "environment_plot/environment_heatmap.pdf",

width = 7,

height = 7)

####calculate correlation between gutmicrobiome and environment

cor_value <-

cor(x = t(as.matrix(environment_expression_data)),

y = t(as.matrix(gutmicrobiome_expression_data1)),

method = "spearman")

cor_value <-

cor_value %>%

as.data.frame() %>%

tibble::rownames_to_column(var = "from") %>%

tidyr::pivot_longer(-from, names_to = "to", values_to = "cor")

library(plyr)

p_value <-

purrr::map(

as.data.frame(t(cor_value)),

.f = function(x) {

value1 <- as.numeric(environment_expression_data[x[1],])

value2 <- as.numeric(gutmicrobiome_expression_data[x[2],])

cor.test(value1, value2, method = "spearman")$p.value

}

) %>%

unlist()

cor_value <-

data.frame(cor_value, p_value, stringsAsFactors = FALSE)

plot(density(cor_value$p_value))

library(plyr)

cor_value <-

cor_value %>%

plyr::dlply(.variables = .(from)) %>%

purrr::map(

.f = function(x) {

x <- x %>%

dplyr::filter(abs(cor) > 0.9)

fdr <- p.adjust(x$p_value, method = "fdr")

x <-

data.frame(x, fdr, stringsAsFactors = FALSE)

x

}

)

cor_value <-

cor_value %>%

do.call(rbind, .) %>%

as.data.frame()

cor_value <-

cor_value %>%

dplyr::filter(abs(cor) > 0.9 & cor != 1)

dim(cor_value)

save(cor_value, file = "cor_value")

load('cor_value')

cor_value1 <-

cor_value %>%

dplyr::filter(abs(cor) > 0.9 & fdr < 0.05)

dim(cor_value1)

dim(cor_value)

sxtTools::setwd_project()

setwd("data_analysis/environment_gutmicrobiome/environment_gutmicrobiome_plot")

# for (idx in 1:nrow(cor_value1)) {

# cat(idx, " ")

# path1 <- file.path(cor_value1$from[idx])

# dir.create(path1, showWarnings = FALSE)

# temp_data <-

# data.frame(

# date = as.character(gutmicrobiome_sample_info$CollectionDate),

# environment = as.numeric(environment_expression_data[cor_value1$from[idx],]),

# gutmicrobiome = as.numeric(gutmicrobiome_expression_data[cor_value1$to[idx],]),

# stringsAsFactors = FALSE

# )

# plot <-

# temp_data %>%

# ggplot(aes(environment, gutmicrobiome)) +

# geom_point() +

# geom_smooth(method = "lm", color = "skyblue") +

# ggrepel::geom_label_repel(aes(x = environment, gutmicrobiome, label = date)) +

# labs(

# x = paste("Exposome (Biological): ", cor_value1$from[idx], sep = ""),

# y = paste("gutmicrobiome: " , cor_value1$to[idx]),

# sep = ""

# ) +

# theme_bw() +

# theme(

# axis.title = element_text(size = 13),

# axis.text = element_text(size = 12),

# plot.background = element_rect(fill = "transparent", color = NA),

# panel.background = element_rect(fill = "transparent", color = NA)

# ) +

# annotate(

# geom = "text",

# x = -Inf,

# y = Inf,

# label = paste(

# "Correlation: ",

# round(cor_value1$cor[idx], 2),

# "\nFDR adjusted P value: ",

# round(cor_value1$fdr[idx], 3),

# sep = ""

# ),

# vjust = 2,

# hjust = -1

# )

#

# name <- paste(cor_value1$from[idx], "_",

# cor_value1$to[idx], ".pdf", sep = "")

#

# ggsave(

# plot,

# filename = file.path(path1, name),

# width = 7,

# height = 7,

# bg = "transparent"

# )

#

# }

sxtTools::setwd_project()

setwd("data_analysis/environment_gutmicrobiome")

cor_value1$from %>% unique()

###correlation network for environment biological and gutmicrobiome

cor_value1$from %>% unique() %>% length()

cor_value1$to %>% unique() %>% length()

library(igraph)

library(ggraph)

library(tidygraph)

###network for all the environment biological and gutmicrobiome

edge_data <-

cor_value1 %>%

# dplyr::filter(from %in% cluster1) %>%

dplyr::rename(from = from,

to = to,

Correlation = cor) %>%

dplyr::mutate(fdr = -log(fdr, 10))

node_data <-

cor_value1 %>%

# dplyr::filter(from %in% cluster1) %>%

dplyr::rename(from = from, to = to) %>%

dplyr::select(from, to) %>%

tidyr::pivot_longer(cols = c(from, to),

names_to = "class",

values_to = "node") %>%

dplyr::mutate(class1 = case_when(

stringr::str_detect(class, "from") ~ "Exposome biological",

TRUE ~ "Gut microbiome"

)) %>%

dplyr::select(node, class1) %>%

dplyr::rename(Class = class1) %>%

dplyr::distinct(node, .keep_all = TRUE)

node_data <-

node_data %>%

dplyr::arrange(Class)

temp_data <-

tidygraph::tbl_graph(nodes = node_data,

edges = edge_data,

directed = TRUE) %>%

dplyr::mutate(Degree = centrality_degree(mode = 'all'))

pal <-

wesanderson::wes_palette(name = "Zissou1", n = 100, type = "continuous")

plot1 <-

ggraph(temp_data,

layout = 'linear',

circular = TRUE) +

geom_edge_arc(aes(color = Correlation),

show.legend = TRUE) +

geom_node_point(aes(fill = Class,

size = Degree),

shape = 21,

show.legend = TRUE) +

scale_fill_manual(

values = c(

"Exposome biological" = ggsci::pal_d3()(10)[5],

"Gut microbiome" = ggsci::pal_d3()(10)[6]

)

) +

geom_node_text(

aes(

x = x * 1.05,

y = y * 1.05,

label = node,

hjust = 'outward',

angle = -((-node_angle(x, y) + 90) %% 180) + 90,

size = 3,

colour = Class

),

size = 3,

alpha = 1,

show.legend = FALSE

) +

guides(

edge_width = guide_legend(title = "-log10(FDR adjusted P value)",

override.aes = list(shape = NA)),

edge_color = ggraph::guide_edge_colorbar(title = "Spearman correlation"),

fill = guide_legend(

title = "Class",

override.aes = list(size = 4, linetype = "blank")

),

size = guide_legend(title = "Degree", override.aes = list(linetype = 0))

) +

ggraph::scale_edge_color_gradientn(colours = pal) +

ggraph::scale_edge_width(range = c(0.1, 1)) +

scale_size_continuous(range = c(0.3, 3)) +

theme_void() +

theme(

plot.background = element_rect(fill = "transparent", color = NA),

panel.background = element_rect(fill = "transparent", color = NA)

)

plot1

ggsave(

plot1,

filename = "environment_gutmicrobiome_correlation_network.pdf",

width = 8.5,

height = 7,

bg = "transparent"

)

ggsave(

plot1,

filename = "environment_gutmicrobiome_correlation_network.png",

width = 8.5,

height = 7,

bg = "transparent"

)

##avoid source

no_function()

##load data

sxtTools::setwd_project()

library(tidyverse)

rm(list = ls())

load("data_analysis/exposomeChemical_environment/temp")

load("data_analysis/exposomeChemical_environment/diff")

temp1 = temp

diff1 = diff

load("data_analysis/exposomeBiological_environment/temp")

load("data_analysis/exposomeBiological_environment/diff")

temp2 = temp

diff2 = diff

load("data_analysis/exposomeChemical_exposomeBiological/temp")

load("data_analysis/exposomeChemical_exposomeBiological/diff")

temp3 = temp

diff3 = diff

load("data_analysis/exposomeChemical_environment/cor_value")

exposomeChemical_environment_cor <-

cor_value %>%

dplyr::filter(abs(cor) > 0.9 & p_value < 0.05)

load("data_analysis/exposomeBiological_environment/cor_value")

exposomeBiological_environment_cor <-

cor_value %>%

dplyr::filter(abs(cor) > 0.9 & p_value < 0.05)

load("data_analysis/exposomeChemical_exposomeBiological/cor_value")

exposomeChemical_exposomeBiological_cor <-

cor_value %>%

dplyr::filter(abs(cor) > 0.9 & p_value < 0.05)

head(exposomeBiological_environment_cor)

head(exposomeChemical_environment_cor)

head(exposomeChemical_exposomeBiological_cor)

cor_value <-

rbind(

exposomeBiological_environment_cor,

exposomeChemical_environment_cor,

exposomeChemical_exposomeBiological_cor

)

###load variable_info

load("data_20200511/exposome/variable_info")

exposomeChemical_variable_info <-

variable_info

load("data_20200511/microbiome/dna_variable_info")

exposomeBiological_variable_info <-

dna_variable_info

load("data_20200511/environment/variable_info")

environment_variable_info <-

variable_info

sxtTools::setwd_project()

setwd("data_analysis/exposome_cloud/")

library(igraph)

library(ggraph)

library(tidygraph)

###plot show sample matching

temp_edge1 = diff1 %>%

dplyr::mutate(

from = paste("exposomeChemical", exposomeChemical.date, sep = "_"),

to = paste("environment", environment.date, sep = "_")

) %>%

dplyr::select(from, to)

temp_node1 =

data.frame(

node = c(

paste(diff1$environment.class, diff1$environment.date, sep = "_"),

paste(

"exposomeChemical",

diff1$exposomeChemical.date,

sep = "_"

)

),

true_name = c(diff1$environment.date,

diff1$exposomeChemical.date),

Class = c(diff1$environment.class, diff1$exposomeChemical.class)

)

temp_edge2 = diff2 %>%

dplyr::mutate(

from = paste("exposomeBiological", exposomeBiological.date, sep = "_"),

to = paste("environment", environment.date, sep = "_")

) %>%

dplyr::select(from, to)

temp_node2 =

data.frame(

node = c(

paste("environment", diff1$environment.date, sep = "_"),

paste(

"exposomeBiological",

diff2$exposomeBiological.date,

sep = "_"

)

),

true_name = c(diff2$environment.date,

diff2$exposomeBiological.date),

Class = c(diff2$environment.class, diff2$exposomeBiological.class)

)

temp_edge3 = diff3 %>%

dplyr::mutate(

from = paste("exposomeBiological", microbiome.date, sep = "_"),

to = paste("exposomeChemical", exp.date, sep = "_")

) %>%

dplyr::select(from, to)

temp_node3 =

data.frame(

node = c(

paste("exposomeChemical", diff3$exp.date, sep = "_"),

paste(

"exposomeBiological",

diff3$microbiome.date,

sep = "_"

)

),

true_name = c(diff3$exp.date,

diff3$microbiome.date),

Class = c(diff3$exp.class, diff3$microbiome.class)

)

value <-

c(

"Environment" = ggsci::pal_d3()(10)[1],

"Exposome (chemical)" = ggsci::pal_d3()(10)[2],

"Metabolome" = ggsci::pal_d3()(10)[3],

"Proteome" = ggsci::pal_d3()(10)[4],

"Exposome (biological)" = ggsci::pal_d3()(10)[5],

"Gut microbiome" = ggsci::pal_d3()(10)[6],

"Blood test" = ggsci::pal_d3()(10)[7],

"Cytokine" = ggsci::pal_d3()(10)[8],

"Toxins and carcinogens" = ggsci::pal_d3()(10)[9]

)

temp_edge =

rbind(temp_edge1,

temp_edge2,

temp_edge3) %>%

dplyr::distinct(.keep_all = TRUE)

temp_node =

rbind(temp_node1,

temp_node2,

temp_node3) %>%

dplyr::distinct(.keep_all = TRUE)

temp_node$Class[temp_node$Class == "exp"] = "exposomeChemical"

temp_node$Class[temp_node$Class == "microbiome"] = "exposomeBiological"

temp_node$Class[temp_node$Class == "exposomeChemical"] = "Exposome (chemical)"

temp_node$Class[temp_node$Class == "exposomeBiological"] = "Exposome (biological)"

temp_node$Class[temp_node$Class == "environment"] = "Environment"

temp_node =

temp_node %>%

dplyr::distinct(.keep_all = TRUE)

match_graph <-

tidygraph::tbl_graph(nodes = temp_node,

edges = temp_edge,

directed = FALSE) %>%

dplyr::mutate(Degree = centrality_degree(mode = 'all'))

plot =

ggraph(match_graph,

layout = 'linear',

circular = TRUE) +

geom_edge_diagonal(show.legend = TRUE) +

geom_node_point(aes(fill = Class, size = Degree),

shape = 21,

show.legend = TRUE) +

scale_fill_manual(values = value) +

scale_color_manual(values = value) +

geom_node_text(

aes(

x = x * 1.05,

y = y * 1.05,

label = true_name,

hjust = 'outward',

angle = -((-node_angle(x, y) + 90) %% 180) + 90,

size = 3,

colour = Class

),

size = 3,

alpha = 1,

show.legend = FALSE

) +

guides(

fill = guide_legend(

title = "Class",

override.aes = list(size = 7, linetype = "blank")

),

size = guide_legend(title = "Degree", override.aes = list(linetype = 0))

) +

scale_size_continuous(range = c(6, 10)) +

theme_void() +

theme(

plot.background = element_rect(fill = "transparent", color = NA),

panel.background = element_rect(fill = "transparent", color = NA)

)

plot

# ggsave(plot, filename = "match_graph.pdf", width = 19, height = 7)

######exposome cloud

edge_data <-

cor_value %>%

dplyr::rename(from = from,

to = to,

Correlation = cor) %>%

dplyr::mutate(fdr = -log(fdr, 10))

node_data <-

cor_value %>%

dplyr::select(from, to) %>%

tidyr::pivot_longer(cols = c(from, to),

names_to = "class",

values_to = "node") %>%

dplyr::mutate(

class1 = case_when(

node %in% exposomeChemical_variable_info$peak_ID ~ "Exposome (chemical)",

node %in% exposomeBiological_variable_info$variable_id ~ "Exposome (biological)",

node %in% environment_variable_info$variable_id ~ "Environment"

)

) %>%

dplyr::select(node, class1) %>%

dplyr::rename(Class = class1) %>%

dplyr::distinct(node, .keep_all = TRUE) %>%

dplyr::arrange(Class) %>%

dplyr::left_join(exposomeChemical_variable_info[, c("peak_ID", "MetabID")],

by = c("node" = "peak_ID")) %>%

dplyr::mutate(true_name = case_when(!is.na(MetabID) ~ MetabID,

TRUE ~ node)) %>%

dplyr::select(node, Class, true_name)

value <-

c(

"Environment" = ggsci::pal_d3()(10)[1],

"Exposome (chemical)" = ggsci::pal_d3()(10)[2],

"Metabolome" = ggsci::pal_d3()(10)[3],

"Proteome" = ggsci::pal_d3()(10)[4],

"Exposome (biological)" = ggsci::pal_d3()(10)[5],

"Gut microbiome" = ggsci::pal_d3()(10)[6],

"Blood test" = ggsci::pal_d3()(10)[7],

"Cytokine" = ggsci::pal_d3()(10)[8],

"Toxins and carcinogens" = ggsci::pal_d3()(10)[9]

)

node_data$true_name <-

node_data$true_name %>%

stringr::str_replace("genus_", "")

edge_data =

edge_data %>%

dplyr::left_join(node_data, by = c("from" = "node")) %>%

dplyr::rename(from_Class = Class, from_true_name = true_name) %>%

dplyr::left_join(node_data, by = c("to" = "node")) %>%

dplyr::rename(to_Class = Class, to_true_name = true_name)

###output node data and edge data

library(openxlsx)

wb = createWorkbook()

modifyBaseFont(wb, fontSize = 12, fontName = "Arial Narrow")

addWorksheet(wb, sheetName = "Node information", gridLines = TRUE)

addWorksheet(wb, sheetName = "Edge information", gridLines = TRUE)

freezePane(wb, sheet = 1, firstRow = TRUE, firstCol = TRUE)

freezePane(wb, sheet = 2, firstRow = TRUE, firstCol = TRUE)

writeDataTable(wb, sheet = 1, x = node_data,

colNames = TRUE, rowNames = FALSE)

writeDataTable(wb, sheet = 2, x = edge_data %>% dplyr::select(from, to, everything()),

colNames = TRUE, rowNames = FALSE)

saveWorkbook(wb, "exposome_cloud.xlsx", overwrite = TRUE)

exposome_cloud <-

tidygraph::tbl_graph(nodes = node_data,

edges = edge_data,

directed = TRUE) %>%

dplyr::mutate(Degree = centrality_degree(mode = 'all'))

pal <-

wesanderson::wes_palette(name = "Zissou1", n = 100, type = "continuous")

plot <-

ggraph(exposome_cloud,

layout = 'linear',

circular = TRUE) +

geom_edge_arc(aes(color = Correlation),

show.legend = TRUE) +

geom_node_point(aes(fill = Class,

size = Degree),

shape = 21,

show.legend = TRUE) +

scale_fill_manual(values = value) +

scale_color_manual(values = value) +

geom_node_text(

aes(

x = x * 1.05,

y = y * 1.05,

label = true_name,

hjust = 'outward',

angle = -((-node_angle(x, y) + 90) %% 180) + 90,

size = 3,

colour = Class

),

size = 3,

alpha = 1,

show.legend = FALSE

) +

guides(

edge_width = guide_legend(title = "-log10(FDR adjusted P value)",

override.aes = list(shape = NA)),

edge_color = ggraph::guide_edge_colorbar(title = "Spearman correlation"),

fill = guide_legend(

title = "Class",

override.aes = list(size = 7, linetype = "blank")

),

size = guide_legend(title = "Degree", override.aes = list(linetype = 0))

) +

ggraph::scale_edge_color_gradientn(colours = pal) +

ggraph::scale_edge_width(range = c(0.2, 2)) +

scale_size_continuous(range = c(3, 15)) +

theme_void() +

theme(

plot.background = element_rect(fill = "transparent", color = NA),

panel.background = element_rect(fill = "transparent", color = NA)

)

plot

# ggsave(

# plot,

# filename = "exposome_cloud_network.pdf",

# width = 8.5,

# height = 7,

# bg = "transparent"

# )

edge_data2 <-

edge_data %>%

dplyr::left_join(exposomeChemical_variable_info[,1:2], by = c("from" = "peak_ID")) %>%

dplyr::mutate(true_name =

case_when(

is.na(MetabID) ~ from,

TRUE ~ MetabID

)) %>%

dplyr::select(true_name, everything()) %>%

dplyr::select(-MetabID)

# write.csv(edge_data2, "edge_data.csv", row.names = FALSE)

# write.csv(node_data, "node_data.csv", row.names = FALSE)

# save(exposome_cloud, file = "exposome_cloud")

dim(edge_data)

dim(node_data)

table(node_data$Class)

sxtTools::setwd_project()

load("data_20200511/environment/expression_data")

environment_expression_data <- expression_data

load("data_20200511/environment/sample_info")

environment_sample_info <- sample_info

load("data_20200511/environment/variable_info")

environment_variable_info <- variable_info

head(environment_variable_info)

value <-

c(

"Environment" = ggsci::pal_d3()(10)[1],

"Exposome (chemical)" = ggsci::pal_d3()(10)[2],

"Metabolome" = ggsci::pal_d3()(10)[3],

"Proteome" = ggsci::pal_d3()(10)[4],

"Exposome (biological)" = ggsci::pal_d3()(10)[5],

"Gut microbiome" = ggsci::pal_d3()(10)[6],

"Blood test" = ggsci::pal_d3()(10)[7],

"Cytokine" = ggsci::pal_d3()(10)[8],

"Toxins and carcinogens" = ggsci::pal_d3()(10)[9]

)

sxtTools::setwd_project()

setwd("data_analysis/exposome_cloud/")

plot <-

igraph::vertex_attr(graph = exposome_cloud) %>%

do.call(cbind, .) %>%

as.data.frame() %>%

dplyr::filter(Class != "Environment") %>%

dplyr::mutate(Degree = as.numeric(Degree)) %>%

dplyr::filter(Degree > 1) %>%

dplyr::arrange(Class, Degree) %>%

dplyr::mutate(true_name = factor(true_name, levels = true_name)) %>%

ggplot(aes(y = true_name, x = Degree)) +

geom_segment(aes(y = true_name, yend = true_name, x = 0, xend = Degree,

color = Class), show.legend = FALSE) +

geom_point(aes(fill = Class), shape = 21,

size = 8,

show.legend = FALSE) +

scale_fill_manual(values = value) +

scale_color_manual(values = value) +

labs(y = "", x = "Degree") +

geom_text(aes(y = true_name, x = Degree + 1,

label = true_name)

# angle = 90, hjust = 1

) +

scale_x_continuous(expand = expansion(mult = c(0, 0.2))) +

theme_bw() +

theme(

panel.grid = element_blank(),

axis.ticks.y = element_blank(),

axis.text = element_text(size = 12),

axis.text.y = element_blank(),

panel.background = element_rect(fill = "transparent", color = NA),

plot.background = element_rect(fill = "transparent", color = NA)

)

plot

# ggsave(plot, filename = "degree_distributation.pdf", width = 7, height = 7)

##get the subnetwork with degree >= 4

idx <-

match(c("Tricholoma", "Cylindrobasidium", "Piriformospora",

"Diisononyl phthalate", "Butylated triphenyl phosphate"),

igraph::vertex_attr(graph = exposome_cloud, name = "true_name"))

name <-

igraph::vertex_attr(graph = exposome_cloud, name = "node")[idx]

name <-

edge_data %>%

dplyr::filter(from %in% name | to %in% name) %>%

dplyr::select(from, to)

name <- unique(c(name$from, name$to))

library(igraph)

idx <- match(name, igraph::vertex_attr(graph = exposome_cloud, name = "node"))

subnetwork <-

igraph::induced_subgraph(graph = exposome_cloud, v = idx)

plot <-

ggraph(subnetwork,

layout = 'kk',

circular = FALSE) +

geom_edge_link(aes(color = Correlation),

show.legend = FALSE) +

geom_node_point(aes(fill = Class,

size = Degree),

shape = 21,

show.legend = FALSE) +

scale_fill_manual(values = value) +

scale_color_manual(values = value) +

geom_node_text(

aes(

x = x * 1.05,

y = y * 1.05,

label = true_name,

# hjust = 'outward',

# angle = -((-node_angle(x, y) + 90) %% 180) + 90,

size = 3,

colour = Class

),

size = 3,

alpha = 1,

show.legend = FALSE

) +

guides(

edge_width = guide_legend(title = "-log10(FDR adjusted P value)",

override.aes = list(shape = NA)),

edge_color = ggraph::guide_edge_colorbar(title = "Spearman correlation"),

fill = guide_legend(

title = "Class",

override.aes = list(size = 7, linetype = "blank")

),

size = guide_legend(title = "Degree", override.aes = list(linetype = 0))

) +

ggraph::scale_edge_color_gradient2(low = ggsci::pal_aaas()(n=10)[1],

mid = "white",

high = ggsci::pal_aaas()(n=10)[2]) +

ggraph::scale_edge_width(range = c(0.2, 2)) +

scale_size_continuous(range = c(3, 15)) +

theme_void() +

theme(

plot.background = element_rect(fill = "transparent", color = NA),

panel.background = element_rect(fill = "transparent", color = NA)

)

plot

# ggsave(

# plot,

# filename = "exposome_cloud_subnetwork.pdf",

# width = 10,

# height = 7,

# bg = "transparent"

# )
